# Root coverage stability: A systematic overview of controlled clinical trials with at least 5 years of follow‐up

**DOI:** 10.1002/cre2.395

**Published:** 2021-02-09

**Authors:** Kristina Bertl, Loukia M. Spineli, Khalid Mohandis, Andreas Stavropoulos

**Affiliations:** ^1^ Department of Periodontology, Faculty of Odontology University of Malmö Malmö Sweden; ^2^ Division of Oral Surgery University Clinic of Dentistry, Medical University of Vienna Vienna Austria; ^3^ Midwifery Research and Education Unit, Hannover Medical School Hannover Germany; ^4^ Division of Conservative Dentistry and Periodontology University Clinic of Dentistry, Medical University of Vienna Vienna Austria; ^5^ Division of Regenerative Dentistry and Periodontology University Clinics of Dental Medicine (CUMD), University of Geneva Geneva Switzerland

**Keywords:** gingival recession, long‐term outcome, muco‐gingival surgery, root coverage, systematic review

## Abstract

**Objectives:**

To systematically assess the long‐term outcome (≥5 years) of root coverage procedures reported in controlled clinical trials.

**Material and Methods:**

Literature search was performed according to the PRISMA guidelines with the following eligibility criteria: (a) English or German language; (b) controlled (CT) or randomised controlled clinical trials (RCT); (c) root coverage procedure with ≥5 years follow‐up; and (d) clinical treatment effect size and/or patient‐related outcome measures (PROMs) reported.

**Results:**

Four CT and 14 RCT with a follow‐up of 5–20 years fulfilled the eligibility criteria; sample size per study ranged from 8 to 70 patients contributing with 18–149 sites. Coronally advanced flap (CAF) and CAF + connective tissue graft (CTG) were the prevalent treatments (i.e., in 24 and 38% of the groups, respectively), while other flap designs and adjuncts (i.e., enamel matrix derivative, bone graft, collagen membrane) were represented only once. For single Miller class I/II gingival recessions (GR), CAF + CTG appeared advantageous compared to other techniques, and provided low residual recession depths (i.e., ≤0.5 mm), and complete root coverage in ≥2/3 of the patients; similar tendency was observed for multiple GR. No data on Miller class III/IV GR is available. No meta‐analysis was feasible due to lack of similarity in the clinical and methodological characteristics across the trials and observed comparisons of interventions.

**Conclusions:**

CAF + CTG appears to be the ‘gold standard’ technique for the treatment of single and multiple Miller class I/II GR also in regard to long‐term (i.e., ≥5 years of follow‐up) treatment outcomes. There is little information regarding the performance, on the long‐term, of other techniques and adjuncts.

## 1 BACKGROUND

The most frequent reason for patients to undergo a cosmetic dentistry treatment (AACD, 2013), which includes periodontal plastic surgeries, was ‘to improve physical attractiveness and self‐esteem’. Taking the costs and morbidity associated with periodontal plastic surgeries into account, it is important that treatments provide a stable and aesthetical long‐term outcome. Various techniques [e.g., coronally advanced flap (CAF), tunnel technique (TUN), laterally positioned flap (LPF), etc.)], flap designs (e.g., CAF with/without vertical releasing incisions, TUN with/without split‐flap, etc.), adjuncts [e.g., free gingival graft (FGG), connective tissue graft (CTG), enamel matrix derivatives (EMD), soft tissue substitutes (STS), membranes for guided tissue regeneration (GTR), etc.], and endless combinations thereof have been introduced for the treatment of gingival recessions (GR), that is, to achieve root coverage. Numerous systematic reviews and consensus reports (AlSarhan et al., 2019; Cairo et al., 2014; Chambrone, Ortega, et al., 2019; Chambrone & Tatakis, 2015; Cheng et al., 2015; Graziani et al., 2014; Moraschini & Barboza, 2016; Tatakis et al., 2015; Tavelli et al., 2018; Tavelli, Barootchi, Cairo, et al., 2019; Tonetti et al., 2014) have been conducted during recent years, reflecting the increased interest in plastic periodontal surgical procedure in both the profession and the patients (Madianos et al., 2016). Most systematic reviews conclude that CAF + CTG should be considered as the ‘gold standard’ for single Miller class I and II GR, also coined as recession type (RT) 1 (Cairo, 2017; Cairo et al., 2014; Chambrone, de Castro Pinto, & Chambrone, 2019; Chambrone, Ortega, et al., 2019; Chambrone & Tatakis, 2015; Tatakis et al., 2015; Tonetti et al., 2014); significantly less evidence is available for multiple GR and GR with interdental attachment loss (i.e., Miller class III and IV, respectively RT 2 and 3; Cairo, 2017; Chambrone, de Castro Pinto, & Chambrone, 2019; Chambrone, Ortega, et al., 2019; Chambrone & Tatakis, 2015; Graziani et al., 2014; Tatakis et al., 2015; Tonetti et al., 2014). The outcome of treatment is determined by the surgical technique and precision (e.g., flap design/tension/thickness/positioning, micro‐surgical approach, etc.; Baldi et al., 1999; Cairo, 2017; Cairo, Cortellini, et al., 2016; Chambrone & Tatakis, 2015; Dodge et al., 2018; Pini Prato et al., 2000; Pini Prato et al., 2005; Skurska et al., 2015; Tatakis et al., 2015; Zucchelli et al., 2019), but also by site‐specific [e.g., initial depth and width of the GR, tooth location, gingival thickness, papilla height, keratinised tissue width (KTW), frenula, etc.] (Cairo, 2017; Chambrone, de Castro Pinto, & Chambrone, 2019; Cortellini & Bissada, 2018; Huang et al., 2005; Pini Prato, Franceschi, et al., 2018; Pini Prato, Magnani, & Chambrone, 2018; Pini‐Prato et al., 2012; Rasperini et al., 2020; Saletta et al., 2001; Tatakis et al., 2015; Zucchelli et al., 2018; Zucchelli et al., 2019), and patient‐related (e.g., smoking status, compliance, oral hygiene habits, etc.; Cairo, 2017; Chambrone, Ortega, et al., 2019; Chambrone & Tatakis, 2015; Tatakis et al., 2015) parameters.

Recently, two systematic reviews (Dai et al., 2019; Tavelli, Barootchi, Cairo, et al., 2019) addressed the question of time on the stability of root coverage procedures. One of them (Dai et al., 2019) summarised the available literature with at least 24 months follow up until July 2018. Based on primarily pairwise meta‐analyses (i.e., short‐ vs. long‐term and comparisons of different techniques) the results indicated that mean root coverage (RC) worsened over time for CAF, but not for CAF + CTG; further, the complete root coverage (CRC) rate and KTW was significantly higher at the long‐term outcome for CAF + CTG compared to CAF only. CAF + EMD displayed no significant changes in terms of CRC rate comparing short‐ versus long‐term results. In the other review (Tavelli, Barootchi, Cairo, et al., 2019) the effect of time (but not specifically the long‐term effect) was assessed by means of network meta‐analyses (NMA), including all studies presenting data for at least two different time points (e.g., after 3 and 12 months). The authors concluded that primarily CTG‐based procedures appeared sufficient to achieve stable results over time, while flap only or flap with the addition of EMD or STS showed a tendency for relapse. Unfortunately, all studies presenting data with a follow‐up >80 months (i.e., four studies in total) were excluded from this specific analysis. In this context, the 10th European Workshop on Periodontology (Tonetti et al., 2014) and the recently updated Cochrane systematic review (Chambrone, Ortega, et al., 2019), have advocated that ‘long‐term’ should be considered as having at least 5 years follow‐up. Hence, no comprehensive summary of long‐term outcomes of root coverage procedures (i.e., ≥5 years), specifically, is existing so far, but it seems relevant, considering the relatively high number of recently published individual long‐term studies (Barootchi et al., 2019; de Santana et al., 2019; Kroiss et al., 2019; Petsos et al., 2020; Tavelli, Barootchi, Di Gianfilippo, et al., 2019).

Thus, the present systematic review aimed to address the following focused question according to the Population, Intervention, Comparison, Outcomes, Study Design (PICOS) criteria (Miller & Forrest, 2001): ‘In patients with single or multiple GR, what is comparatively the long‐term outcome (≥ 5 years) of root coverage procedures with a flap alone or flap with adjuncts (soft tissue grafts or substitutes, bone grafts or substitutes, membranes, or biologic agents) and/or different flap designs in terms of clinical outcome parameters?’. Further, the aim was to provide a hierarchy of interventions by means of NMA, wherever possible.

## 2 MATERIALS AND METHODS

### 2.1 Protocol and eligibility criteria

The present systematic review was reported according to the criteria of the Preferred Reporting Items for Systematic Reviews and Meta‐analyses (PRISMA; Appendix 1; Liberati et al., 2009; Moher et al., 2009) and was registered at PROSPERO (CRD42020165024). The following inclusion criteria were applied during the literature search: (a) English or German language; (b) prospective interventional studies [i.e., controlled (CT) or randomised controlled clinical trials (RCT)]; (c) root coverage procedure with ≥5 years follow‐up; (d) clinical treatment effect size and/or patient‐related outcome measures (PROMs) reported; and (e) full‐text available.

### 2.2 Information sources and literature search

Electronic search was performed in three sources (last search 29 April 2020; no date restriction used): Medline (PubMed), Scopus, and CENTRAL (Ovid). The database Medline (Pubmed) was searched with the following keywords: (gingival recession OR gingival recessions OR root exposure OR gingival dehiscence OR mucosal recession OR soft tissue dehiscence OR gingival defect) AND (root coverage OR plastic surgery OR muco‐gingival surgery OR mucogingival surgery). For the other 2 databases comparable terms were used but modified to be suitable for specific criteria of the particular database. Additionally, grey literature (conference abstracts and www.opengrey.eu) was browsed and a ‘manual‐search’ through the electronically available material of relevant journals, including publications ahead of print, was performed: *Journal of Clinical Periodontology, Journal of Periodontology*, *Journal of Periodontal Research*, *Clinical Oral Investigations*, *Journal of Dental Research*, and *Parodontologie*; screening of the reference lists of previous reviews and selected full‐texts was also conducted. Finally, a forward search via Science Citation Index of included papers was added and ClinicalTrials.gov was checked on unpublished or on‐going studies.

### 2.3 Data collection and extraction

Two authors (K.B., K.M.) independently checked title, abstract, and finally full‐text on the pre‐defined eligibility criteria. Abstracts with unclear methodology or follow‐up were included in full‐text assessment to avoid exclusion of potentially relevant articles. One author (K.B.) repeated the literature search. Kappa scores regarding agreement on the articles to be included in the full‐text analysis and those finally chosen were calculated. In case of ambiguity, consensus through discussion was achieved together with a third author (A.S.).

Two authors (K.B., K.M.) extracted twice the following data at baseline (i.e., before surgery; BL), at an intermediate time‐point (IM) [i.e., after 6 or preferably 12 months if existing], and at final evaluation (i.e., ≥ 60 months; FE): recession depth (mm; RD), RD reduction (i.e., BL to FE), RD stability (i.e., IM to FE), CRC (%), CRC stability (i.e., IM to FE), mean RC (%), RC stability (i.e., IM to FE), KTW (mm), KTW increase (i.e., BL to FE), KTW stability (i.e., IM to FE), and probing pocket depth (PD). Further, any evaluation of the aesthetic outcome and/or PROMs, study design, sample size, patient/tooth/GR characteristics, type of intervention, and evaluation time‐points were recorded. Finally, a list of potential predictors for the outcome in general and its stability on the long‐term was created and its frequency of reporting in each paper extracted: gingival phenotype/thickness, GR width, flap details (i.e., incision design, positioning), CTG details (i.e., donor region, harvesting technique, CTG thickness and coverage), root conditioning, details on any cervical lesion [i.e., detectability of the cemento‐enamel junction (CEJ), absence/presence of a cervical step, restoration of any cervical step], timepoint of suture removal, and details on the supportive periodontal treatment provided during the follow‐up (i.e., interval, surveillance of oral hygiene habits).

### 2.4 Risk of bias assessment

Two authors (K.B., K.M.) independently evaluated the risk of bias (RoB) of the studies eligible for NMA applying the Cochrane Collaboration's Tool for assessing RoB Version 2 [Cochrane Handbook for Systematic Reviews of Interventions; (Sterne et al., 2019)]. The following domains were evaluated as ‘low risk’, ‘high risk’, or ‘some concerns’ risk: (a) randomisation process; (b) deviations from intended interventions; (c) missing outcome data; (d) measurement of the outcome; and (e) selection of the reported results. The overall risk of bias for an individual study was judged as: ‘low risk’, if all criteria were evaluated to be of low risk; ‘high risk’, if at least one criterion was evaluated to be of high risk; ‘some concerns’, if at least one criterion was evaluated to provide some concerns but no criterion with the judgement high risk. One author (K.B.) repeated the assessment and in case of ambiguity consensus through discussion with another author (A.S.) was achieved. Additionally, any report on any funding (e.g., self‐supported, research grant, industry, etc.) was collected.

### 2.5 Synthesis of results

Two primary outcome parameters (i.e., RD and CRC at FE) and several secondary outcome parameters (i.e., RD reduction, RD stability, CRC stability, RC at FE, RC stability, KTW at FE, KTW increase, KTW stability) were defined for statistical analysis. If necessary, outcome parameters were calculated (e.g., RD reduction by subtracting RD at FE from RD at BL, RD stability by subtracting RD at FE from RD at IM, etc.) and/or the authors of the original publications were contacted. Aesthetic outcome parameters, PD at FE, PROMs, and the potential predictors were summarised for overview in tables.

### 2.6 Statistical analysis

**FIGURE 1:**
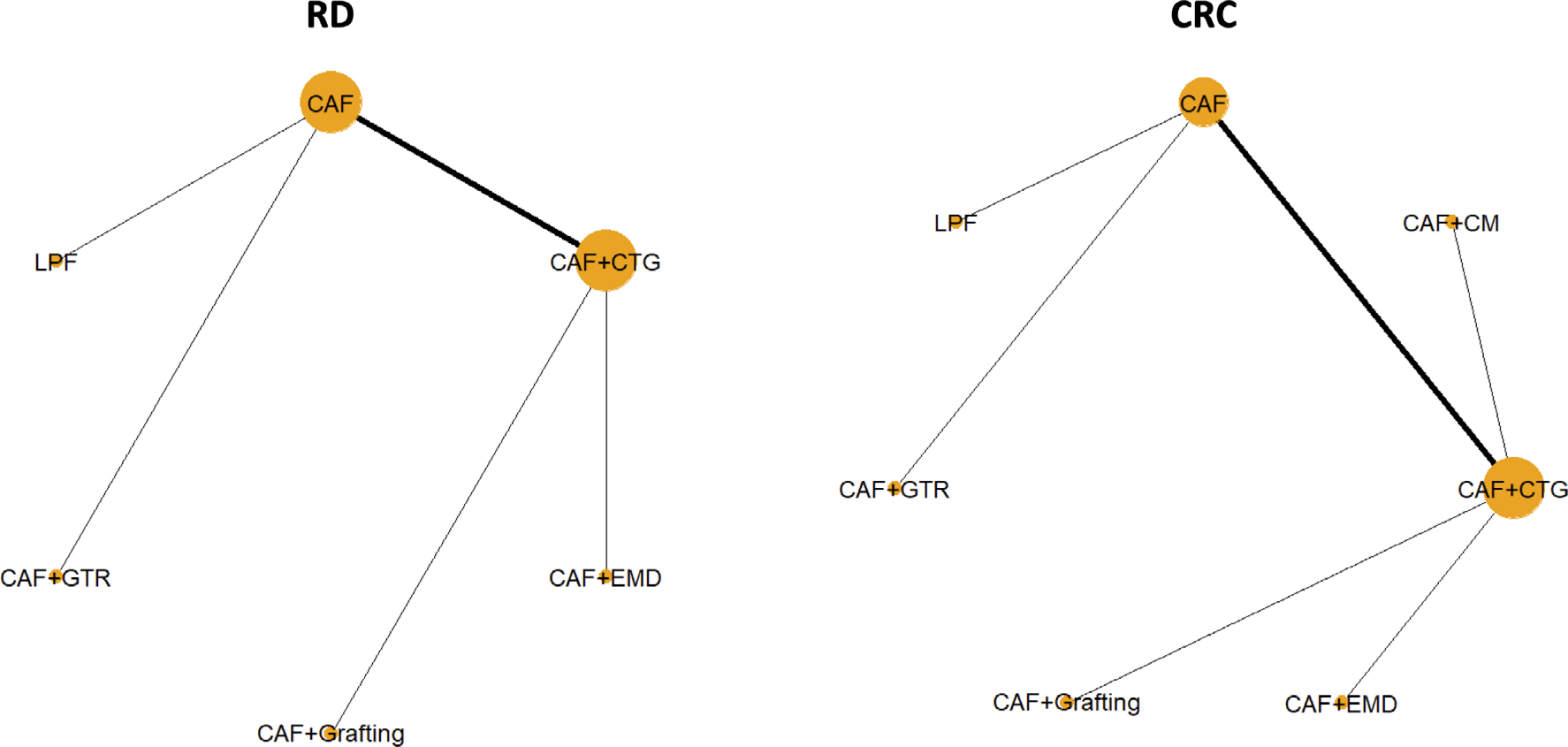
A panel of network plots for the primary outcomes RD and CRC. The nodes refer to the interventions and the lines that link the nodes indicate the observed comparisons. The size of the nodes is proportional to the number of comparisons that include the node. The thickness of the lines is proportional to the number of trials that investigate the corresponding comparison. CRC, complete root coverage; RD, recession depth

For statistical analysis only RCT fitting to one of the following four groups were considered eligible: (a) single Miller class I and/or II (RT 1) GR, (b) single Miller class III and/or IV (RT 2 and 3) GR, (c) multiple Miller class I and/or II (RT 1) GR, (d) multiple Miller class III and/or IV (RT 2 and 3) GR. Hence, CT and studies including patients with single and multiple GR and/or Miller class I/II and III/IV, were not considered as comparable. All outcomes were measured using the mean difference, except for CRC, which was measured using the odds ratio in the logarithmic scale (log OR). NMA was intended for each outcome; for details see Appendix 2 (including Figure 1 and Appendix 3).

## 3 RESULTS

### 3.1 Study selection

The flowchart of the literature search is presented in Appendix 4. Kappa scores regarding agreement on the articles to be included in the full‐text analysis and those finally chosen were 0.85 and 1.0, respectively (*p* < .001). Out of 3275 identified studies, 50 articles were selected for full‐text review; 32 trials were excluded for various reasons (Appendix 5). Finally, 4 CT (Dominiak et al., 2006; Francetti et al., 2018; Kroiss et al., 2019; Pini‐Prato et al., 2010) and 14 RCT (Barootchi et al., 2019; de Santana et al., 2019; Kuis et al., 2013; Leknes et al., 2005; McGuire et al., 2012; McGuire et al., 2014; McGuire & Scheyer, 2016; Moslemi et al., 2011; Paolantonio et al., 1997; Petsos et al., 2020; Pini Prato et al., 2011; Rasperini et al., 2018; Tavelli, Barootchi, Di Gianfilippo, et al., 2019; Zucchelli et al., 2014) were included herein; one RCT (Nickles et al., 2010) was excluded as the follow‐up at a later timepoint was included (Petsos et al., 2020). No ongoing, unpublished studies were identified.

### 3.2 Study characteristics

An overview of study design, sample size, patient/tooth/GR characteristics, type of intervention, and evaluation time‐points is given in Table 1.

**TABLE 1:** Characteristics of the included studies in relation to the gingival recession type

Study	Study design	**No. of patients (BL/FE)** **No. of teeth (BL/FE)**	**Diagnosis** **Age range (mean)** **m/f** **Systemic condition** **Smoking status**	**Intervention (n of teeth at FE)** **Group I** **Group II** **Group III**	**Single/multiple recessions** **Minimum recession depth** **Maxilla/mandible (n of teeth)** **Incisor/canine/premolar/molar (*n*)**	Evaluation timepoints (*m*)
**Single gingival recessions with Miller class I/II**						
Leknes et al. (2005)	RCT, SM	20/11 40/22	Miller's class I & II BL: NR (38.4) BL: 10/10 Healthy BL: 8 smokers	CAF (11 teeth) CAF + GTR (biodegradable membrane; Guidor; 11 teeth)	22/0 ≥3 mm NR but both included NR but only canines and premolars	12, 72
Pini‐Prato et al. (2011)	RCT, SM	10/9 20/18	Miller's class I & II BL: 25 to 57 (33.6) BL: 2/8 Healthy BL: 2 mokers	CAF (root surface polishing; 9 teeth) CAF (root planing; 9 teeth)	18/0 ≥2 mm 18/0 BL: 5/7/8/0	3, 12, 60, 168
McGuire et al. (2012)	RCT, SM	17/9 34/18	Miller's class I & II 44 to 74 (55.4) 4/5 5 healthy, 4 reported generalised anxiety‐related symptoms 0 smokers, 2 former smokers	CAF + CTG (9 teeth) CAF + EMD (9 teeth)	18/0 ≥4 mm NR but both included NR but only incisors, canines and premolars	12, 120
Kuis et al. (2013)	RCT, SM	37/37 114/114	Miller's class I & II 20 to 52 (31.1) 12/25 Healthy 0 smokers	CAF (57 teeth) CAF + CTG (57 teeth)	114/0 NR 96/18 22/26/58/8	6, 12, 24, 60
McGuire et al. (2014)	RCT, SM	30/20 60/40	Miller's class II 29 to 68 (52.5) 3/17 Healthy 0 smokers	CAF + CTG (20 teeth) CAF + ß‐tricalcium phosphate with recombinant human PDGF‐BB (20 teeth)	40/0 ≥3 mm 36/4 Mainly canines (30) and no molars	6, 60
McGuire and Scheyer (2016)	RCT, SM	25/17 50/34	Miller's class I & II 18 to 70 (51.3) 5/12 Healthy BL: 13 former smokers	CAF + CTG (17 teeth) CAF + CM (Mucograft; 17 teeth)	34/0 ≥3 mm BL: 20/5 NR but molars excluded	6, 60
Rasperini et al. (2018)^a^	RCT, PG	85/25 85/25	Miller's class I & II 37 to 63 (group I: 51.1; group II: 47.4) 10/15 Healthy 3 smokers	CAF (13 teeth) CAF + CTG (12 teeth)	25/0 ≥2 mm 25/0 1/13/11/0	6, 12, 108
Francetti et al. (2018)	CT, PG	20/20 20/20	Miller's class I & II NR (group I: 32.8; group II: 34.2) 9/11 Healthy 5 smokers	CAF (10 teeth) CAF + CTG (10 teeth)	20/0 ≥2 mm 20/0 NR but molars excluded	12, 36, 60
de Santana et al. (2019)	RCT, PG	36/32 36/32	Miller's class I BL: NR (34) BL: 10/26 Healthy 0 smokers	CAF (16 teeth) LPF (16 teeth)	32/0 NR 32/0 BL: 7/19/10/0	12, 60
**Multiple gingival recessions with Miller class I/II**						
Zucchelli et al. (2014)	RCT, PG	50/50 149/149	Miller's class I & II 22 to 46 (group I: 34.2; group II: 33.2) 21/29 Healthy NR but smokers with ≤10 cigarettes per day could be included	CAF (73 teeth) CAF + CTG (76 teeth)	0/149 ≥2 mm 149/0 37/44/68/0	6, 12, 60
Kroiss et al. (2019)	CT, PG	39/32 233/NR	Miller's class I & II^b^ BL: 7/32 BL: 24 to 69 (group I: 43.6; group II: 46.6) Healthy NR but former smokers (>6 months) could be included	CAF + CTG (NR) CAF + ADMA (Tutoplast Dermis Allograft Tissue Matrix; NR)	BL: 0/233 NR BL: 168/70^c^ BL: 39/61/90/48^c^	6, 60
Tavelli, Barootchi, Di Gianfilippo, et al. (2019)	RCT, PG	24/19 80/67	Miller's class I & II NR (BL: 52.1) 7/12 Healthy 0 smokers	CAF + ADMA (Alloderm; 33 teeth) TUN + ADMA (Alloderm; 34 teeth)	0/67 ≥2 mm (on at least 1 tooth) 67/0 NR but molars excluded	6, 144
**Multiple gingival recessions with Miller class I/II/III**						
Pini‐Prato et al. (2010)	CT, SM	13/13 93/93	Miller's class I, II & III 24 to 51 (31.4) 3/10 Healthy 3 smokers	CAF (49 teeth) CAF + CTG (44 teeth)	0/93 NR 93/0 25/23/41/4	6, 12, 60
**Single and multiple gingival recessions mixed with Miller class I/II**						
Paolantonio et al. (1997)	RCT, PG	70/70 70/70	Miller's class I & II 25 to 48 (31.8) 32/38 Healthy NR	FGG (35 teeth) CAF + CTG (35 teeth)	Single & multiple recessions were included but only a single recession contributed to the study NR NR NR but molars excluded	60
Dominiak et al. (2006)	CT, PG	52/37 NR/98	Miller's class I & II 17 to 53 (30.3) 10/27 Healthy NR	DPBF (33 teeth) CAF + CTG (41 teeth) CAF + GTR (collagen membrane; 24 teeth)	38/60 >2 mm 64/34 22/43/33/0	12, 24, 60
Moslemi et al. (2011)	RCT, SM	16/15 32/30	Miller's class I & II^d^ 24 to 45 (39.4) 7/8 Healthy 0 smokers but former smokers could be included	CAF + CTG (15 teeth) CAF + ADMA (Alloderm; 15 teeth)	Single and multiple recessions were included but only a single recession contributed to the study ≥2 mm NR NR but molars excluded	6, 60
Barootchi et al. (2019)	RCT, PG	20/17 NR/29^e^	Miller's class I & II BL: 20 to 60 (42.6) BL: 8/12 Healthy 0 smokers	CAF + CTG (16 teeth) CAF + CTG with an EC (13 teeth)	NR ≥2 mm BL: 12/8 BL: 0/6/14/0	6, 144
Petsos et al. (2020)	RCT, unclear	15/8 38/23	Miller's class I & II 29 to 45 (34.0) 4/5 NR 1 smoker, 1 former smoker	Envelope pouch + CTG (10 teeth) CAF + GTR (biodegradable membrane; Guidor; 13 teeth)	16/7 ≥3 mm 17/7 0/20/4/0	3, 120, 240

*Note*: Presented data are based on the population/teeth at final evaluation unless indicated otherwise.

Abbreviations: ADMA, acellular dermal matrix allograft; BL, baseline; CAF, coronally advanced flap; CM, collagen matrix; CTG, connective tissue graft; CT, controlled clinical trial; DPBF, double pedicle bilateral flap; EC, epithelial collar; EMD, enamel matrix derivative; FE, final evaluation; f, female; FGG, free gingival graft; GTR, guided tissue regeneration; LPF, laterally positioned flap; m, male; m, months; NR, not reported; PDGF‐BB, platelet‐derived growth factor‐BB; PG, parallel group; RCT, randomised controlled clinical trial; RD, recession depth; SM, split mouth; TUN, tunnel technique.

^a^ The study was initiated as multi‐centre study, but for the long‐term outcome only the patients of one specific centre were reported (no loss to follow‐up for this specific centre).

^b^ Treatment of the multiple recessions included also Miller's class III or IV, but in the statistical analysis only Miller's class I & II had been included.

^c^ Inconsistencies in the number of teeth included at baseline is due to inconsistencies in the original publication.

^d^ The CAF + CTG group also included Miller's class III, but in the statistical analysis only Miller's class I & II had been included.

^e^ Authors report in the publication additionally on 14 adjacent sites, which had been treated with CAF only, but these sites have not been included herein.

#### 3.2.1 Study populations

The sample size in the various studies ranged from 8 to 70 patients, contributing with 18 to 149 sites; the number of patients and sites at FE was always reported, except for a single study (Kroiss et al., 2019). All participants were judged as healthy or at least as not having any systemic disease that could interfere with periodontal tissue healing; one study (Petsos et al., 2020) did not report on any systemic conditions. Six studies included only non‐smokers, 8 studies mixed (former) smokers and non‐smokers, and 4 studies did not report in detail on the smoking status. All studies reported loss of study subjects to follow‐up, ranging from 0 to 15 patients among studies; one study (Rasperini et al., 2018), which had been originally a multi‐centre study (Cortellini et al., 2009), reported the long‐term outcome of only one specific centre (i.e., 25 out of original 85 patients).

#### 3.2.2 Type of intervention

The following root coverage procedures were included among the 18 studies with a follow‐up period ranging from 60 to 240 months:

- CAF (9 groups)
- CAF + CTG (13 groups) & CAF + CTG with an epithelial collar (EC; 1 group)
- CAF + acellular dermal matrix allograft (ADMA; 3 groups)
- CAF + GTR (3 groups)
- CAF + EMD (1 group)
- CAF + grafting (1 group)
- CAF + collagen matrix (CM; 1 group)
- Envelope pouch + CTG (1 group)
- FGG (1 group)
- Double pedicle bilateral flap (DPBF; 1 group)
- LPF (1 group)
- TUN + ADMA (1 group)

In one publication (Barootchi et al., 2019), the authors reported additionally on 14 sites, adjacent to the CAF + CTG test sites, that had been treated with CAF only; these sites were not considered herein as separate/individual CAF group.

Except for a single study (Dominiak et al., 2006) including three intervention groups, all studies compared two different interventions; nine studies each had a parallel group or a split‐mouth design. Five studies (Francetti et al., 2018; Kuis et al., 2013; Pini‐Prato et al., 2010; Rasperini et al., 2018; Zucchelli et al., 2014) reported on the comparison CAF versus CAF + CTG, two studies (Kroiss et al., 2019; Moslemi et al., 2011) on the comparison CAF + CTG versus CAF + ADMA, while all other comparisons appeared only once:

- CAF + CTG versus CAF + CTG with an EC (Barootchi et al., 2019)
- DPBF versus CAF + CTG versus CAF + GTR (Dominiak et al., 2006)
- CAF versus CAF + GTR (Leknes et al., 2005)
- CAF + CTG versus CAF + EMD (McGuire et al., 2012)
- CAF + CTG versus CAF + grafting (McGuire et al., 2014)
- CAF + CTG versus CAF + CM (McGuire & Scheyer, 2016)
- FGG versus CAF + CTG (Paolantonio et al., 1997)
- CAF with root planing versus CAF with root surface polishing (Pini Prato et al., 2011)
- CAF versus LPF (de Santana et al., 2019)
- CAF + ADMA versus TUN + ADMA (Tavelli, Barootchi, Di Gianfilippo, et al., 2019)
- Envelope pouch + CTG versus CAF + GTR (Petsos et al., 2020)

#### 3.2.3 Description of defect and site characteristics

Most of the studies (*n* = 15) included Miller class I or II GR (Miller, 1985), while one study each included only Miller class I GR, only Miller Class II GR, or mixed Miller class I, II, and III GR. Nine studies reported on the outcome of root coverage procedures for single GR, four studies for multiple GR, five studies mixed single and multiple GR in their analysis; for one (Barootchi et al., 2019) of the latter studies the no. of single and multiple GR was unclear. Most of the studies (*n* = 12) defined ≥/>2 mm or ≥3 mm RD as inclusion criterion and one study (McGuire et al., 2012) included only GR with ≥4 mm, while the other five studies did not specify. The exact location of the GR (i.e., upper or lower jaw, incisors/canines/premolars/molars) including the exact amount per location was given in 10 studies, while the other 8 studies lacked either information on the exact numbers and/or on tooth type, jaw type, or both.

### 3.3 Reported outcome variables and treatment effect size

All primary and secondary outcome parameters as well as PD at FE of the individual studies are displayed in Tables 2 and 3. The aesthetic outcome parameters and PROMs are summarised in Table 4 and the reporting frequency of potential predictors on the outcome is displayed in Appendix 6 and 7.

**TABLE 2:** Clinical outcomes parameters at baseline and final evaluation in relation to the gingival recession type

**Study (year)** **Study design**	Intervention	BL		Follow‐up period (m)	FE				
		RD (mm)	KTW (mm)		RD (mm)	CRC (%)	RC (%)	KTW (mm)	PD (mm)
**Single gingival recessions with Miller class I/II**									
Leknes et al. (2005) RCT	CAF	3.80 ± 1.2	2.60 ± 0.5	72	2.50 ± 1.4	9.1	NR	2.60 ± 0.7	1.00 ± 0.6
	CAF + GTR	4.00 ± 1.0	2.60 ± 0.7		2.60 ± 1.5	18.2	NR	2.60 ± 0.9	1.40 ± 0.6
Pini‐Prato et al. (2011) RCT	CAF (root surface polishing)	3.10 ± 1.1^a^	3.10 ± 1.3^a^	168	0.90 ± 1.2	56.0	NR	2.40 ± 1.8	1.00 ± 0.6
	CAF (root planing)	2.90 ± 1.0^a^	2.70 ± 1.2^a^		0.90 ± 0.9	33.0	NR	2.30 ± 1.3	1.00 ± 0.5
McGuire et al. (2012) RCT	CAF + CTG	4.00 ± 0.5	2.56 ± 0.7	120	0.33 ± 1.0	77.8	89.8 ± 22.7	4.00 ± 0.7	1.56 ± 0.8
	CAF + EMD	4.00 ± 0.0	2.67 ± 0.7		0.67 ± 0.9	55.6	83.3 ± 21.7	3.56 ± 1.1	1.89 ± 0.9
Kuis et al. (2013) RCT	CAF	2.63 ± 0.8	1.33 ± 1.2	60	0.46 ± 0.6	59.6	82.7 ± 23.8	2.25 ± 0.8	NR
	CAF + CTG	2.63 ± 0.7	1.33 ± 1.2		0.19 ± 0.4	82.5	92.3 ± 19.2	2.7 ± 0.6	NR
McGuire et al. (2014) RCT	CAF + CTG	3.40 ± 0.6	2.05 ± 0.9	60	0.35 ± 0.8	75.0	89.4 ± 21.6	3.68 ± 1.0	2.63 ± 0.6
	CAF + grafting	3.25 ± 0.6	2.03 ± 0.7		0.90 ± 1.1	60.0	74.1 ± 37.3	3.03 ± 0.8	2.50 ± 0.6
McGuire and Scheyer (2016) RCT	CAF + CTG	3.20 ± 0.4^a^	2.78 ± 1.4^a^	60	NR	88.2	95.5 ± 12.8	4.12 ± 0.9	1.50 ± 0.5
	CAF + CM	3.14 ± 0.2^a^	2.44 ± 1.0^a^		NR	52.9	77.6 ± 29.2	3.41 ± 1.1	1.65 ± 0.5
Rasperini et al. (2018) RCT	CAF	3.80 ± 0.6	3.80 ± 1.5	108	1.00 ± 0.8	38.5	65.5 ± 35.7	3.60 ± 0.7	1.40 ± 0.3
	CAF + CTG	3.80 ± 0.8	3.20 ± 1.0		0.50 ± 0.5	66.7	81.9 ± 27.1	4.80 ± 0.7	1.50 ± 0.3
Francetti et al. (2018) CT	CAF	2.90 ± 1.0	2.89 ± 1.1	60	1.15 ± 1.1	60.0	65.7 ± 32.2	2.89 ± 0.8	1.63 ± 1.3
	CAF + CTG	2.70 ± 0.5	2.30 ± 0.8		0.44 ± 0.6	70.0	85.4 ± 20.8	3.00 ± 0.8	1.61 ± 0.8
de Santana et al. (2019) RCT	CAF	3.20 ± 0.5	1.50 ± 1.6	60	0.50 ± 0.6	56.3	82.1	1.70 ± 0.6	1.60 ± 0.6
	LPF	3.40 ± 0.6	1.30 ± 1.8		0.30 ± 0.6	68.8	91.6	4.70 ± 1.5	1.30 ± 0.5
**Multiple gingival recessions with Miller class I/II**									
Zucchelli et al. (2014) RCT	CAF	3.05 ± 0.9	1.43 ± 0.5	60	0.30 ± 0.7	78.1	92.4 ± 14.4	2.75 ± 0.7	1.10 ± 0.3
	CAF + CTG	3.15 ± 1.0	1.47 ± 0.5		0.09 ± 0.3	90.8	97.6 ± 7.7	3.18 ± 0.7	1.22 ± 0.4
Kroiss et al. (2019) CT	CAF + CTG	2.84 ± 0.8^a^	1.69 ± 1.1^a^	60	0.52 ± 0.7	NR	NR	3.98 ± 0.9	1.16 ± 0.6
	CAF + ADMA	2.77 ± 0.8^a^	2.04 ± 0.9^a^		0.92 ± 0.7	NR	NR	3.06 ± 1.0	1.19 ± 0.8
Tavelli et al. (2019) RCT	CAF + ADMA	2.56 ± 1.4	3.09 ± 1.3	144	0.84 ± 0.6	27.3	65.8 ± 21.7	3.39 ± 0.9	1.59 ± 0.5
	TUN + ADMA	2.29 ± 1.0	2.54 ± 1.2		0.91 ± 0.6	29.4	63.6 ± 23.4	2.62 ± 1.6	1.42 ± 0.5
**Multiple gingival recessions with Miller class I/II/III**									
Pini‐Prato et al. (2010) CT	CAF	2.90 ± 1.3	NR	60	0.80 ± 0.8	35.0	70.0 ± 32.0	NR	NR
	CAF + CTG	3.60 ± 1.3	NR		0.40 ± 0.5	52.0	89.0 ± 13.0	NR	NR
**Single and multiple gingival recessions mixed with Miller class I/II**									
Paolantonio et al. (1997) RCT	FGG	3.11 ± 0.3	1.57 ± 0.3	60	1.50 ± 0.4	8.6	53.2 ± 21.5	5.23 ± 0.5	NR
	CAF + CTG	3.43 ± 0.4	1.94 ± 0.4		0.58 ± 0.5	48.6	85.2 ± 17.9	4.75 ± 0.9	NR
Dominiak et al. (2006) CT	DPBF	2.88 ± 0.8	3.36 ± 1.6	60	0.85 ± 1.0	NR	68.9 ± 35.3	3.45 ± 2.4	1.03 ± 1.0
	CAF + CTG	4.54 ± 1.5	1.32 ± 1.3		0.83 ± 1.2	NR	82.8 ± 24.0	4.66 ± 1.3	1.19 ± 1.2
	CAF + GTR	3.79 ± 1.4	3.38 ± 2.0		0.38 ± 1.1	NR	90.0 ± 28.9	4.31 ± 0.9	1.05 ± 1.1
Moslemi et al. (2011) RCT	CAF + CTG	3.33 ± 1.4	1.93 ± 1.3	60	1.83 ± 1.1	13.3	39.8 ± 40.6	2.70 ± 1.2	1.20 ± 0.6
	CAF + ADMA	2.87 ± 0.9	1.90 ± 1.3		1.27 ± 1.0	20.0	54.6 ± 34.9	1.87 ± 1.2	0.86 ± 0.4
Barootchi et al. (2019) RCT	CAF + CTG	2.75 ± 0.9	1.18 ± 0.4	144	0.62 ± 0.5	56.3	74.5 ± 25.1	3.87 ± 0.7	1.43 ± 0.5
	CAF + CTG with EC	2.54 ± 0.7	2.07 ± 0.7		0.57 ± 0.4	61.5	77.7 ± 18.3	3.94 ± 0.5	1.50 ± 0.6
Petsos et al. (2020) RCT	Envelope pouch + CTG	4.10 ± 1.5	2.90 ± 2.2	240	2.60 ± 2.5	14.1	43.6 ± 80.7	6.10 ± 2.2	1.10 ± 0.2
	CAF + GTR	5.20 ± 2.0	1.80 ± 1.9		4.10 ± 2.1	12.5	26.6 ± 44.0	2.60 ± 2.0	1.30 ± 0.7

*Note*: If necessary, outcome parameters were calculated and/or requested from the authors.

Abbreviations: ADMA, acellular dermal matrix allograft; BL, baseline; CAF, coronally advanced flap; CM, collagen matrix; CRC, complete root coverage; CTG, connective tissue graft; CT, controlled clinical trial; DPBF, double pedicle bilateral flap; EC, epithelial collar; EMD, enamel matrix derivative; FE, final evaluation; FGG, free gingival graft; GTR, guided tissue regeneration; KTW, keratinised tissue width; LPF, laterally positioned flap; m, months; NR, not reported; PD, probing pocket depth; RC, mean root coverage; RCT, randomised controlled clinical trial; RD, recession depth; TUN, tunnel technique.

^a^ Baseline values are based on a higher number of patients compared to final evaluation.

**TABLE 3:** Change from baseline to final evaluation and stability after the intermediate time‐point of clinical outcome parameters in relation to the gingival recession type

Study (year) study design	Intervention	Follow‐up period (m)	Change from BL to FE^a^		Stability ‐ Change from IM to FE^b^			
			RD (mm)	KTW (mm)	RD (mm)	CRC (%)	RC (%)	KTW (mm)
**Single gingival recessions with Miller class I/II**								
Leknes et al. (2005) RCT	CAF	72	1.30 ± 1.3	0.00 ± 0.6	−1.00 ± 1.4	NR	NR	−0.40 ± 0.7
	CAF + GTR		1.40 ± 1.3	0.00 ± 0.8	−0.50 ± 1.4	NR	NR	−0.50 ± 0.8
Pini‐Prato et al. (2011) RCT	CAF (root surface polishing)	168	NR	NR	NR	NR	NR	NR
	CAF (root planing)		NR	NR	NR	NR	NR	NR
McGuire et al. (2012) RCT	CAF + CTG	120	3.67 ± 1.1	1.44 ± 0.7	−0.22 ± 0.4	−11.1	−6.5 ± 19.7	0.11 ± 1.1
	CAF + EMD		3.33 ± 0.9	0.89 ± 1.3	−0.44 ± 0.5	−22.2	−11.1 ± 18.8	0.56 ± 1.1
Kuis et al. (2013) RCT	CAF	60	2.17 ± 0.7	0.92 ± 1.0	−0.18 ± 0.6	−14.1	−8.2 ± 21.0	0.04 ± 0.8
	CAF + CTG		2.44 ± 0.6	1.37 ± 1.0	−0.10 ± 0.4	−10.5	−4.9 ± 16.7	0.12 ± 0.6
McGuire et al. (2014) RCT	CAF + CTG	60	3.05 ± 0.9	1.63 ± 1.1	−0.28 ± 0.6	−15.0	−8.9 ± 20.8	0.40 ± 0.8
	CAF + grafting		2.35 ± 1.2	1.00 ± 0.9	−0.55 ± 1.0	−10.0	−15.8 ± 27.0	0.08 ± 0.5
McGuire and Scheyer (2016) RCT	CAF + CTG	60	NR	NR	NR	−5.9	−2.0 ± 8.1	−0.06 ± 1.3
	CAF + CM		NR	NR	NR	−17.7	−11.9 ± 22.5	−0.71 ± 1.2
Rasperini et al. (2018) RCT	CAF	108	2.80 ± 0.7	−0.20 ± 1.3	−0.10 ± 0.7	7.7	−0.8 ± 32.8	0.70 ± 0.6
	CAF + CTG		3.30 ± 0.7	1.60 ± 0.9	0.10 ± 0.5	8.4	2.8 ± 26.9	1.00 ± 0.8
Francetti et al. (2018) CT	CAF	60	1.75 ± 1.0	0.00 ± 0.9	−0.05 ± 1.0	−10.0	3.4 ± 32.1	−0.22 ± 0.7
	CAF + CTG		2.26 ± 0.6	0.70 ± 0.8	0.11 ± 0.7	−10.0	−4.0 ± 18.7	−0.20 ± 1.2
de Santana et al. (2019) RCT	CAF	60	2.70 ± 0.6	0.20 ± 1.4	−0.30 ± 0.5	−32.6	−12.6	−0.20 ± 0.7
	LPF		3.10 ± 0.6	3.40 ± 1.7	−0.10 ± 0.5	−9.0	−4.5	0.30 ± 1.5
**Multiple gingival recessions with Miller class I/II**								
Zucchelli et al. (2014) RCT	CAF	60	2.75 ± 0.8	1.32 ± 0.6	−0.20 ± 0.6	−11.0	−4.9 ± 11.4	0.67 ± 0.6
	CAF + CTG		3.06 ± 0.9	1.71 ± 0.6	0.04 ± 0.3	4.0	1.1 ± 9.9	0.71 ± 0.7
Kroiss et al. (2019) CT	CAF + CTG	60	2.27 ± 0.8	2.27 ± 1.0	−0.45 ± 0.6	NR	NR	0.06 ± 1.2
	CAF + ADMA		1.82 ± 0.7	1.14 ± 1.0	−0.35 ± 0.5	NR	NR	−0.04 ± 1.1
Tavelli et al. (2019) RCT	CAF + ADMA	144	1.72 ± 1.2	0.29 ± 1.6	−0.43 ± 0.6	−25.3	−22.8 ± 27.2	0.50 ± 1.5
	TUN + ADMA		1.38 ± 0.8	0.07 ± 2.0	−0.60 ± 0.6	−21.8	−25.7 ± 26.6	0.61 ± 1.7
**Multiple gingival recessions with Miller class I/II/III**								
Pini‐Prato et al. (2010) CT	CAF	60	2.10 ± 1.2	NR	−0.20 ± 0.7	−2.0	−7.0 ± 29.5	NR
	CAF + CTG		3.20 ± 1.3	NR	0.10 ± 0.5	7.0	2.0 ± 13.0	NR
**Single and multiple gingival recessions mixed with Miller class I/II**								
Paolantonio et al. (1997) RCT	FGG	60	1.61 ± 0.4	3.66 ± 0.4	−0.20 ± 0.7	NR	NR	NR
	CAF + CTG		2.85 ± 0.4	2.81 ± 0.8	0.10 ± 0.5	NR	NR	NR
Dominiak et al. (2006) CT	DPBF	60	2.03 ± 0.9	0.09 ± 2.1	−0.45 ± 0.9	NR	−17.8 ± 31.3	−0.43 ± 2.2
	CAF + CTG		3.71 ± 1.4	3.34 ± 1.3	−0.22 ± 1.1	NR	−6.0 ± 22.5	0.05 ± 1.3
	CAF + GTR		3.42 ± 1.3	0.93 ± 1.8	−0.13 ± 1.0	NR	−1.3 ± 26.5	0.06 ± 1.0
Moslemi et al. (2011) RCT	CAF + CTG	60	1.50 ± 1.4	0.77 ± 1.3	−0.70 ± 0.7	−13.3	−27.1 ± 35.3	−0.03 ± 1.1
	CAF + ADMA		1.60 ± 1.2	−0.03 ± 1.0	−0.97 ± 0.9	−53.3	−33.2 ± 30.5	−1.00 ± 1.2
Barootchi et al. (2019) RCT	CAF + CTG	144	2.13 ± 0.7	2.69 ± 0.6	−0.37 ± 0.4	−25.0	−16.5 ± 31.8	1.25 ± 0.6
	CAF + CTG with EC		1.97 ± 0.6	1.87 ± 0.6	−0.46 ± 0.4	−23.1	−19.4 ± 24.1	0.10 ± 0.6
Petsos et al. (2020) RCT	Envelope pouch + CTG	240	1.50 ± 2.3	3.20 ± 1.0	−1.60 ± 2.1	0.0	−29.0 ± 69.9	0.20 ± 2.3
	CAF + GTR		1.10 ± 2.8	0.80 ± 0.4	−1.20 ± 2.3	0.0	−18.9 ± 38.9	0.90 ± 0.5

*Note*: If necessary, outcome parameters were calculated and/or requested from the authors.

Abbreviations: ADMA, acellular dermal matrix allograft; BL, baseline; CAF, coronally advanced flap; CM, collagen matrix; CRC, complete root coverage; CTG, connective tissue graft; CT, controlled clinical trial; DPBF, double pedicle bilateral flap; EC, epithelial collar; FE, final evaluation; FGG, free gingival graft; GTR, guided tissue regeneration; IM, intermediate time‐point (6 or 12 months); KTW, keratinised tissue width; LPF, laterally positioned flap; m, months; NR, not reported; PD, probing pocket depth; RC, mean root coverage; RCT, randomised controlled clinical trial; RD, recession depth; TUN, tunnel technique.

^a^ Positive values indicate a RD reduction or an increase in KTW.

^b^ Positive values indicate a RD reduction, an increase of KTW, mean RC, or CRC.

**TABLE 4:** Aesthetic outcome parameters and PROMs at final evaluation in relation to the gingival recession type

Study (year)	Intervention	Parameter – Outcome									
**Single gingival recessions with Miller class I/II**											
Pini‐Prato et al. (2011)	CAF (root surface polishing)	Dentin hypersensitivity^a^				50% at BL, 33% at FE					
	CAF (root planing)					40% at BL, 33% at FE					
McGuire et al. (2012)	CAF + CTG	Colour, texture, contour comp. to adjacent tissue^b^	55.6% equivalent texture 66.7% equivalent colour 12.5% equivalent contour		Preference of procedure^c^	6 favoured CAF + EMD (67%) 2 no preference (22%) 1 favoured CAF + CTG (11%)	Satisfaction with outcome^c^	6 no preference (67%) 2 favoured CAF + EMD (22%) 1 favoured CAF + CTG (11%)		Dentin hyper‐sensitivity	3 at FE (33%)
	CAF + EMD		88.9% equivalent texture 88.9% equivalent colour 100% equivalent contour								1 at FE (11%)
McGuire et al. (2014)	CAF + CTG	Colour, texture comp. to adjacent tissue^b^	NS (no data provided)	Satisfaction with outcome^c^	14 very satisfied (70%) 6 satisfied (30%)	Discomfort^c^	NS (no data provided)	Which technique for re‐treatment^c^	NS (no data pro‐vided)	Dentin hyper‐sensitivity	NS (no data provided)
	CAF + grafting				14 very satisfied (70%) 4 satisfied (20%) 1 unsatisfied (5%) 1 very unsatisfied (5%)						
McGuire and Scheyer (2016)	CAF + CTG	Colour, texture comp. to adjacent tissue^b^	82.4% equivalent colour 35.3% equivalent texture, 64.7% more firm			Satisfaction with outcome^c^		10 very satisfied (59%) 6 satisfied (35%) 1 unsatisfied (6%)			
	CAF + CM		88.2% equivalent colour 88.2% equivalent texture					9 very satisfied (53%) 6 satisfied (35%) 2 unsatisfied (12%)			
Rasperini et al. (2018)	CAF	Dentin hypersensitivity				46.2% at BL, 0% at FE					
	CAF + CTG					25% at BL, 0% at FE					
Francetti et al. (2018)	CAF	Dentin hypersensitivity				5 at BL (50%), 2 at FE (20%)					
	CAF + CTG					4 at BL (40%), 1 at FE (10%)					
**Multiple gingival recessions with Miller class I/II**											
Zucchelli et al. (2014)	CAF	Colour match^b^	85.2 (81.6–88.8)^d^		Contour match^b^	76.8 (70.1–83.4)^d^	Keloid formation^b^	4%	Satisfaction (VAS)^c^	82.8 (78.6–87.0)^d^	
	CAF + CTG		73.6 (67.3–79.9)^d^			87.2 (82.8–91.5)^d^		44%		81.6 (76.4–86.7)^d^	
Tavelli et al. (2019)	CAF + ADMA	RES^b^	7.01 ± 1.4	Satisfaction (VAS)^c^	8.67 ± 1.3	Willingness for re‐treatment^c^	100% Yes	Perception of stability (VAS)^c^	No data provided		
	TUN + ADMA		6.93 ± 1.3		8.31 ± 1.4		100% Yes				
**Single and multiple gingival recessions mixed with Miller class I/II**											
Barootchi et al. (2019)	CAF + CTG	RES^b^	7.64 ± 1.4	Satisfaction (VAS)^c^	9.13 ± 1.5	Willingness for re‐treatment^c^	100% Yes	Perception of stability (VAS)^c^	No data provided		
	CAF + CTG with EC		7.42 ± 1.2		8.96 ± 1.3		100% Yes				
Petsos et al. (2020)	Envelope pouch + CTG	Improvement of defect^c^		1 no improvement (10%) 3 satisfied (30%) 1 good (10%) 5 total resolution (50%)			Satisfaction with outcome^c^	8 very good (80%) 1 good (10%) 1 insufficient (10%)			
	CAF + GTR			2 no improvement (15%) 5 satisfied (39%) 2 good (15%) 4 total resolution (31%)				4 very good (31%) 3 good (23%) 1 average (8%) 2 sufficient (15%) 3 insufficient (23%)			

Abbreviations: ADMA, acellular dermal matrix allograft; BL, baseline; CAF, coronally advanced flap; CM, collagen matrix; CTG, connective tissue graft; DPBF, double pedicle bilateral flap; EC, epithelial collar; EMD, enamel matrix derivative; FE, final evaluation; FGG, free gingival graft; GTR, guided tissue regeneration; LPF, laterally positioned flap; NS, not significant; PROM, patient‐related outcome measures; RES, root coverage aesthetic score (Cairo et al., 2009); TUN, tunnel technique; VAS, visual analogue scale.

^a^ BL and FE data are based on a different number of patients.

^b^ Judged by clinician.

^c^ Judged by patients.

^d^ Mean (95% confidence intervals).

#### 3.3.1 Single Miller class I/II GR: 9 studies (1 CT, 8 RCT)

The following summary focuses primarily on groups treated either by CAF or CAF + CTG, as all other treatment groups were represented only once. Overall, among the single GR the mean RD at FE ranged from 0.19 to 2.6 mm and CRC was achieved in 9.1–88.2% of the patients. However, by excluding a single study (Leknes et al., 2005) the mean residual RD was ≤1.15 mm and CRC was maintained in at least every third patient. All groups treated by CAF + CTG resulted in a mean residual RD ≤0.5 mm (corresponding to a RC rate >80%) and achieved CRC in at least 2/3 of the patients. In comparison mean residual RD in the groups treated by CAF only ranged from 0.46 to 1.15 mm with a single group (Leknes et al., 2005) reporting a high mean residual RD of 2.5 mm. Mean PD at FE remained <2 mm except for a single study (McGuire et al., 2014) showing a mean PD of approximately 2.5 mm. Mean KTW increase ranged from −0.2 to 3.4 mm with only a single group (CAF) resulting in a minor mean loss of 0.2 mm and the KTW of all groups treated by CAF + CTG increased on average by ≥0.7 mm. In terms of stability after the IM timepoint the mean RD worsened by ≤0.55 mm except for a single group (CAF; Leknes et al., 2005) with a mean increase of RD by 1.0 mm, while two groups (Francetti et al., 2018; Rasperini et al., 2018) treated by CAF + CTG presented at FE even a lower mean residual RD compared to IM. CRC among the CAF + CTG groups was reduced by ≤15% with one group achieving even an increase by 8.4%, while the CAF only groups presented a bigger range from 7.7% increase to 32.6% reduction.

#### 3.3.2 Multiple Miller class I/II GR: 3 studies (1 CT, 2 RCT)

Overall, among the multiple Miller class I/II GR the mean RD at FE ranged from 0.09 to 0.92 mm and CRC was achieved in 27.3–90.8% of the patients. In the only study (Zucchelli et al., 2014) comparing CAF versus CAF + CTG the mean residual RD was reduced by 2/3 by the adjunct use of a CTG (i.e., 0.3 vs. 0.09 mm), but both groups achieved a RC and CRC rate >75 and >90%, respectively; however, only in the group treated by CAF + CTG the mean residual RD and the RC and CRC rate improved from IM to FE. A second group with CTG as adjunct (Kroiss et al., 2019) achieved also a low mean residual RD (i.e., 0.52 mm), while the groups with ADMA as adjunct presented a mean residual RD ≥0.84 mm and CRC in less than 1/3 of the patients. In one study (Tavelli, Barootchi, Di Gianfilippo, et al., 2019) comparing the results with ADMA and either CAF or TUN the average increase in KTW was minor compared to the other studies (i.e., ≤0.29 vs. ≥1.14 mm) and the patients lost about 20–25% in RC and CRC rate from IM to FE. Mean PD at FE remained for all study groups <1.6 mm.

#### 3.3.3 Multiple Miller class I/II/III GR: 1 CT

Only a single study (Pini‐Prato et al., 2010) comparing CAF versus CAF + CTG combined multiple Miller class I, II, and III GR. Mean residual RD was 50% less (i.e., 0.8 vs. 0.4 mm) in the group treated by CAF + CTG with a RC rate of 89%; however, CRC at FE was achieved in both groups (CAF and CAF + CTG) in only 35 and 52% of the patients, respectively. Nevertheless, in the group treated by CAF + CTG the mean residual RD and the RC and CRC rate improved from IM to FE.

#### 3.3.4 Single and multiple Miller class I/II GR: 5 studies (1 CT, 4 RCT)

Overall, in this relatively heterogenic group due to inclusion of both single and multiple GR the mean RD at FE ranged from 0.38 to 4.1 mm and CRC was achieved in 8.6–61.5% of the patients. Except for a single study (Moslemi et al., 2011) with a relatively high mean residual RD in the CAF + CTG group (i.e., RD 1.83 mm, RC 39.8%), the mean residual RD remained in the CAF + CTG groups ≤0.83 mm with a RC rate ≥75%. In this specific study (Moslemi et al., 2011), the CAF + ADMA group achieved superior results compared to the CAF + CTG group, but mean residual RD was still high with 1.27 mm and RC and CRC rate low with 54.6 and 20%, respectively. One of the CAF + CTG groups (Paolantonio et al., 1997) presented an improved (i.e., lower) mean residual RD in the long‐term follow‐up (i.e., comparing IM to FE), while the above‐mentioned group treated by CAF + ADMA (Moslemi et al., 2011) lost almost 1 mm in RD in the follow‐up after IM, which resulted also in a loss of 33.2 and 53.3% in the RC and CRC rate, respectively. Mean PD at FE remained for all study groups ≤1.5 mm.

For the following groups no single CT/RCT with a long‐term outcome was available:

- Single Miller class III/IV GR
- Multiple Miller class III/IV GR

#### 3.3.5 Aesthetic outcome parameters and PROMs

Altogether, 10 out of the 18 studies reported either aesthetic outcome parameters and/or PROMs (Table 4). Based on these data it appears that procedures with CTG as adjunct might be less favourable in terms of colour, texture, and contour compared to the adjacent tissue, in terms of keloid formation, and in terms of patients' preference of the procedure, but patient satisfaction with the outcome was not affected and remained high (>80% in VAS). Further, dentin hypersensitivity showed in general an improvement, but 100% success should not be expected.

#### 3.3.6 Reporting frequency of potential predictors on the outcome

Some of the potential predictors were often (i.e., in ≥50% of the studies) not reported: gingival phenotype/thickness, GR width, graft thickness, detectability of the CEJ, presence of a cervical step, restoration of the cervical step, and long‐term surveillance of oral hygiene habits. While other parameters were given in almost every study (i.e., maximum missing in one of the included studies): KTW, CAF incision design, flap positioning in relation to the CEJ, and root conditioning.

### 3.4 Synthesis of results

**FIGURE 2:**
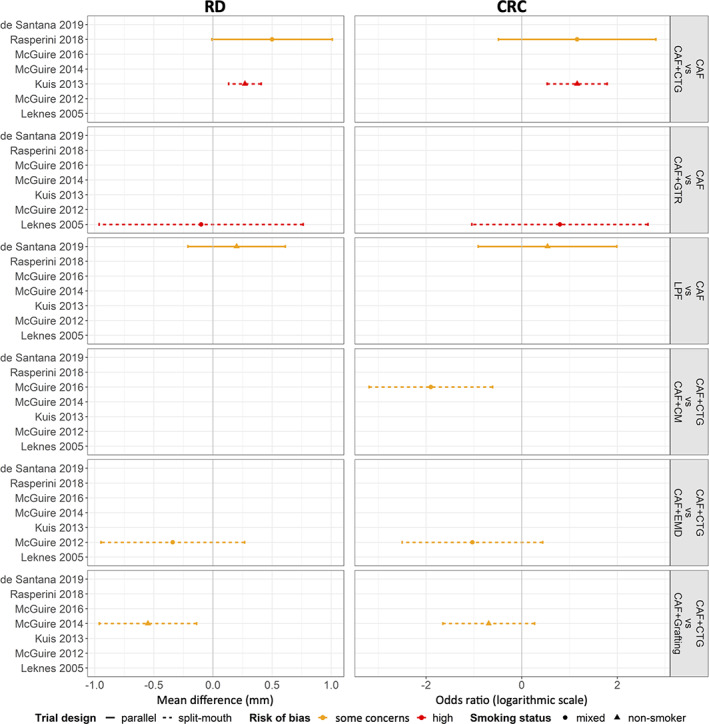
A panel of forest plots for all observed comparisons in RD and CRC. The unique observed comparisons and the included trials appear on the right and the left of the panel, respectively. The trials have been ordered chronologically. The *x*‐axis refers to the mean difference and the log OR for the corresponding primary outcomes RD and CRC, respectively. The design of the trial (parallel group vs. split‐mouth design), the level of RoB (some concerns vs. high), and the smoking status of the participants (mixed vs. non‐smoker) are indicated with different line types (solid vs. dashed), colours (orange vs. red), and point shapes (circle vs. triangle), respectively. The vertical grey line above zero implies no difference between the compared interventions. A positive mean difference and log OR indicate that the second intervention in the comparison is more favourable. CRC, complete root coverage; RD, recession depth

**FIGURE 3:**
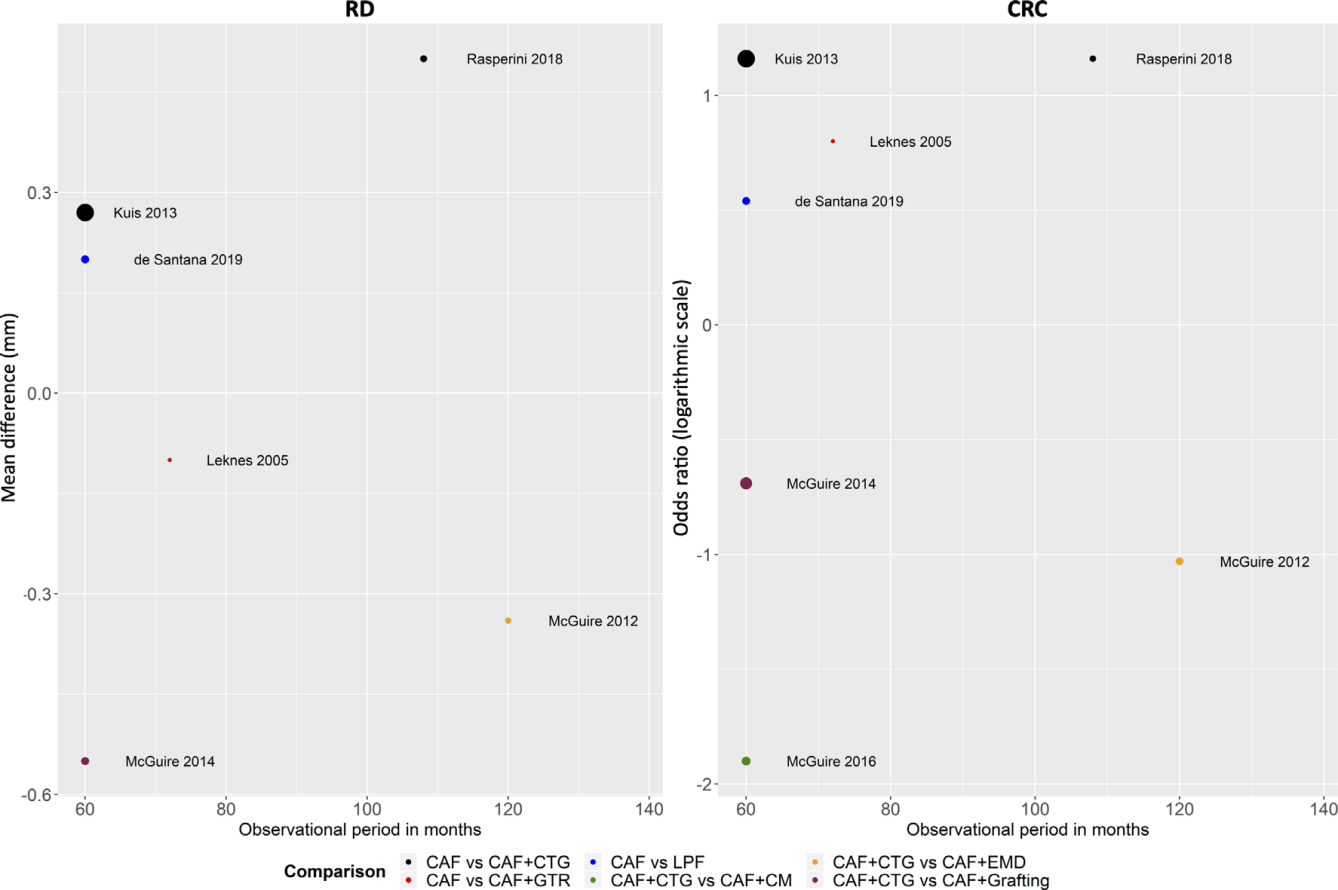
Bubble plots that illustrate the relationship between the observational period in months (*x*‐axis) and the relative treatment effect observed in each trial (*y*‐axis) for the primary outcome parameters RD and CRC. Each point refers to a trial. The size of each point has been weighted by the inverse of the variance of the corresponding trial: the smaller the trial, the smaller the size of the point and vice versa. Different colours refer to the comparisons investigated in each trial. A positive mean difference and log OR indicate that the second intervention in the comparison is more favourable. CRC, complete root coverage; RD, recession depth

Eight RCT (de Santana et al., 2019; Kuis et al., 2013; Leknes et al., 2005; McGuire et al., 2012; McGuire et al., 2014; McGuire & Scheyer, 2016; Pini Prato et al., 2011; Rasperini et al., 2018) reported on the treatment outcomes of single Miller class I/II GR. Only two RCT (Tavelli, Barootchi, Di Gianfilippo, et al., 2019; Zucchelli et al., 2014) reported on the treatment outcomes of multiple Miller class I/II GR, including four different types of intervention and could not be compared/pooled. The remaining studies were either not randomised or included a mixture of single and multiple GR. Hence, any attempt for a (N)MA was only possible for single Miller class I/II GR; one (Pini Prato et al., 2011) of the eight studies had to be excluded as it was comparing CAF versus CAF with different methods of root surface modifications. However, due to lack of similarity in clinical and methodological characteristics across the trials and observed comparisons, any synthesis of the trials was not performed. Therefore, the present overview is primarily of descriptive nature. Figure 1 and Appendix 3 illustrate the networks for the primary and secondary outcomes of the study; for details see also Appendix 2. Further, an overview of the results of the seven studies on single Miller class I/II GR is provided by forest plots (Figure 2 for the primary outcome parameters; Appendix 8 for the secondary outcome parameters) and bubble plots (Figure 3 for the primary outcome parameters; Appendix 9 for the secondary outcome parameters).

### 3.5 RoB assessment and funding

Out of the seven studies, which were considered originally for NMA, two studies (Kuis et al., 2013; Leknes et al., 2005) have been judged as presenting high risk and the other five studies (de Santana et al., 2019; McGuire et al., 2012; McGuire et al., 2014; McGuire & Scheyer, 2016; Rasperini et al., 2018) were judged to provide some concerns; the results are summarised in Appendix 10.

Five studies (de Santana et al., 2019; Pini Prato et al., 2011; Pini‐Prato et al., 2010; Rasperini et al., 2018; Zucchelli et al., 2014) have been self‐supported by the authors and did not receive any additional funding, five studies (Barootchi et al., 2019; Kuis et al., 2013; Moslemi et al., 2011; Petsos et al., 2020; Tavelli, Barootchi, Di Gianfilippo, et al., 2019) were supported by a research grant and/or university, three studies (Kroiss et al., 2019; McGuire et al., 2014; McGuire & Scheyer, 2016) were partially supported by the company of the tested product, four studies (Dominiak et al., 2006; Francetti et al., 2018; McGuire et al., 2012; Paolantonio et al., 1997) did not report on any funding, and one study (Leknes et al., 2005) received the product free‐of‐charge, but received otherwise no funding.

## 4 DISCUSSION

The primary aim of GR treatment is CRC, with natural appearance of the tissues, and stability of the outcome on the long‐term. The present systematic review aimed to provide an overview of the available literature on the long‐term outcome of root coverage procedures and to provide – if possible – recommendations on which techniques have the highest probability for a successful outcome on the long‐term. The results of one of the most recent systematic reviews (Chambrone, Ortega, et al., 2019), including studies with short‐term follow‐up provided the following ranking in terms of clinical outcomes and cost‐to‐benefit ratio for single Miller class I/II GR: (a) CAF + CTG, (b) CAF + ADMA, (c) CAF + EMD, (d) CAF + CM, and (e) CAF. Significantly less evidence was available for multiple Miller class I/II GR, but the authors suggested that a similar hierarchical order, with that in single GR, might apply.

Most available controlled clinical, long‐term studies regarded single Miller class I/II GR; that is, one CT and eight RCT with a follow‐up of 5–14 years. Unfortunately, the lack of clinical and methodological homogeneity in the included trials alongside the limited number of trials (Figure 1) to allow for a moderator analysis (that would have adjusted the results for the observed clinical and methodological heterogeneity), rendered the application of NMA and pairwise meta‐analysis not feasible. Therefore, the overview presented herein is primarily of descriptive nature. When excluding the CAF group of a single study (Leknes et al., 2005) with significantly inferior outcomes than what is usually reported (i.e., mean residual RD of about 2.5 mm), the mean residual RD ranged for the CAF and CAF + CTG groups from 0.46 to 1.15 mm and from 0.19 to 0.5 mm, respectively; CRC ranged from 33 to 60% and from 66.7 to 88.2%, respectively. Thus, it seems there is a tendency for more favourable treatment outcomes with CAF + CTG. This is supported by the fact that 2 CAF + CTG groups (Francetti et al., 2018; Rasperini et al., 2018) showed ‘creeping attachment’ over time resulting in a lower mean residual RD at FE compared to IM. In general, except for the above‐mentioned CAF group presenting especially bad outcomes (Leknes et al., 2005), all interventions for treating single GR showed acceptable stability of the outcome, with an increase in RD of ≤0.55 mm, on average, after the IM. Based on the results of the few individual studies, CAF + CTG appeared also superior in the direct comparisons to CAF + EMD (McGuire et al., 2012), to CAF + CM (McGuire & Scheyer, 2016), and to CAF + grafting (McGuire et al., 2014). Only a single RCT (de Santana et al., 2019) assessed another flap design (i.e., LPF) after 5 years showing also low mean residual RD (i.e., 0.3 mm) with high increase in KTW (i.e., 3.4 mm) and a high stability over time.

Regarding the treatment of multiple Miller class I/II (and III) GR, the few available studies showed better outcomes for CAF + CTG compared to only CAF (Pini‐Prato et al., 2010; Zucchelli et al., 2014) or to ADMA (Kroiss et al., 2019). In particular, CAF groups slightly lost over the years from what was originally achieved (i.e., 0.2 mm increase in RD), while the addition of a CTG resulted in a minor improvement from IM to FE (i.e., ‘creeping attachment’ occurred); the mean residual RD for the CAF + CTG group was only 33–50% of the CAF group (Pini‐Prato et al., 2010, Zucchelli et al., 2014). Use of ADMA as an alternative to CTG with CAF (Kroiss et al., 2019; Tavelli, Barootchi, Di Gianfilippo, et al., 2019), yield similar or higher mean residual RD compared to what was reported in other studies for CAF alone and 2‐ to 9‐times higher values compared to CAF + CTG. Additionally, in both studies (Kroiss et al., 2019, Tavelli, Barootchi, Di Gianfilippo, et al., 2019) assessing CAF + ADMA, the CAF + AMDA groups lost about two‐times more in RD over the years compared to what was reported in other studies for CAF only, and no ‘creeping attachment’ was observed. In contrast, one RCT (Moslemi et al., 2011) reported superior results for CAF + ADMA compared to CAF + CTG. However, in this specific study the mean RD at baseline (i.e., prior to intervention) was 0.46 mm higher in the CAF + CTG group and the mean residual RD after intervention was unexpectedly high in the CAF + CTG group (i.e., 1.83 mm; this is about three‐ to five‐times higher than what is reported in most of the other studies included herein); additionally, the CAF + ADMA group showed again no long‐term stability, by losing almost 1 mm in RD from IM to FE. Similarly, in another controlled but non‐randomised study (Dominiak et al., 2006) showing inferior results for CAF + CTG compared to CAF + GTR, again the CAF + CTG group presented with a mean RD at baseline being 0.75 mm higher compared to the CAF + GTR group.

In perspective, the lack of long‐term data of controlled clinical trials with at least 5 years follow‐up for several interventions and combination approaches (i.e., TUN + CTG, CAF + EMD, CAF + CM, CAF + CTG + EMD, etc.) has to be kept in mind. However, taking also non‐prospective and/or shorter follow‐ups into account, a 6‐year retrospective analysis (Bhatavadekar et al., 2019) indicated comparable results for the comparison CAF + CTG versus TUN + CTG with the results being within the range of the RCTs (Pini‐Prato et al., 2010, Zucchelli et al., 2014) included herein. In terms of combination treatments, 3‐year results of CAF + CTG versus CAF + CTG + EMD have been recently published (Mercado et al., 2020a) indicating some advantages by the addition of EMD. The principle of EMD being advantageous – especially on the long‐term – would be its potential to actually achieve at least partly periodontal regeneration instead of ‘only’ a long junctional epithelium (McGuire et al., 2016). However, due to the extra financial burden for the patient and the overall limited and unclear data on the histological outcome of healing after adjunct use of EMD, future long‐term trials assessing EMD as only or as additional adjunct are warranted.

There was no evidence from controlled clinical trials about the long‐term outcome of treatment of GR with interdental attachment loss (i.e., Miller class III/IV or RT 2/3) – neither for single nor for multiple defects. Nevertheless, there are medium‐term data (i.e., 3 years follow‐up; Cairo et al., 2015; Mercado et al., 2020b) as well as 20‐year case series (Pini Prato, Franceschi, et al., 2018; Pini Prato, Magnani, & Chambrone, 2018) available with the latter probably providing the best long‐term evidence available at the moment with a direct comparison of the outcome of GR without (Miller class I) or with interdental attachment loss (Miller class III) treated either by CAF (Pini Prato, Magnani, & Chambrone, 2018) or CAF + CTG (Pini Prato, Franceschi, et al., 2018). For both interventions the presence of interdental attachment loss significantly reduced the chance to achieve CRC after 1 year. However, while in sites treated with CAF recurrence of GR was significantly associated with the presence of interdental attachment loss, this was not the case for sites treated with CAF + CTG; that is, similar to what was described above for single and multiple GR without interdental attachment loss, the addition of CTG appears to provide better long‐term stability.

In this context, except from the type of surgical intervention, other factors should also be considered as relevant for a successful long‐term outcome. In particular, specific surgical details (e.g., releasing incisions, details on CTG harvesting, root conditioning, etc.), site‐related (e.g., phenotype, GR width, cervical lesions, etc.) and patient‐related factors (e.g., smoking), and performance of supportive periodontal treatment (i.e., frequency, surveillance of oral hygiene habits, etc.) are debated as potentially relevant in achieving and maintaining a successful treatment result and thereby reducing the probability of GR recurrence. Herein it was attempted to give an overview of such frequently discussed parameters and their reporting frequency (Appendix 6 and 7). Regular supportive periodontal treatment, including specifically the close surveillance of oral hygiene habits, is considered as a major determinant of long‐term success (Cairo et al., 2014; Dai et al., 2019; Leknes et al., 2005; McGuire et al., 2014; Moslemi et al., 2011; Pini Prato, Franceschi, et al., 2018; Rasperini et al., 2018; Zucchelli et al., 2014; Zucchelli & De Sanctis, 2005); for example, a horizontal toothbrushing technique increased the risk for a relapse 11‐times (Moslemi et al., 2011). Further, the tooth region appears to affect the probability of achieving CRC (Zucchelli et al., 2018; Zucchelli et al., 2019). Naturally, this cannot be altered by the surgeon, but one should consider assessing different techniques separately in the upper and lower anterior sextant, which have been described as having the best and worst probability for a good outcome, respectively (Zucchelli et al., 2019). Additionally, a low baseline KTW as well as KTW <2 mm after the intervention were reported as negative predictors (Barootchi et al., 2019; Pini Prato et al., 2011; Pini Prato, Franceschi, et al., 2018; Pini Prato, Magnani, & Chambrone, 2018; Pini‐Prato et al., 2012; Tavelli, Barootchi, Cairo, et al., 2019; Tavelli, Barootchi, Di Gianfilippo, et al., 2019), while the gain of KTW might be affected by the chosen technique [e.g., by leaving the CTG exposed or not exposed (Dodge et al., 2018)].

In this context, although CAF + CTG is described as the ‘gold standard’, CTG as an adjunct might not be necessary in every single case; that is, recent studies indicated that a thick tissue at baseline might not need the addition of a CTG (Cairo, Cortellini, et al., 2016; Rasperini et al., 2019, 2020). Hence, one can speculate whether the gingival thickness at the end of the procedure is the main determining factor for long‐term stability and not necessarily the addition of a CTG; for example, in one of the RCT (Tavelli, Barootchi, Di Gianfilippo, et al., 2019) a gingival thickness ≥1.2 mm after 6 months was a significant predictor for stability of the gingival margin later on. However, the gingival phenotype/thickness was not reported in >70% of the studies included herein. Details on the CTG harvesting procedure itself might be another relevant factor (Tavelli, Ravidà, Lin, et al., 2019). Different harvesting techniques (split‐flap procedure or de‐epithelialised CTG) result in different tissue compositions of the graft (Bertl et al., 2015), which might affect the resulting tissue thickness but potentially also the risk for keloid formation. Nevertheless, a large variation in terms of reporting frequency (5.5–95%) of these and other potentially relevant factors was observed (Appendix 6).

Root coverage procedures are primarily cosmetic dentistry and aim to improve patient aesthetics (Cairo, 2017; Cairo, Pagliaro, et al., 2016). Hence, also aesthetic outcomes (based on both, professional evaluation as well as on patient perception) and PROMs should be evaluated. Ten out of 18 studies herein reported on various PROMS, ranging from dentin hypersensitivity to colour/texture/contour match, aesthetic scores, and patients' satisfaction. Although the analysis is again limited to an overview (Table 4), some tendencies can be deducted. Procedures including a CTG harbour the risk to be less favourable in terms of colour, texture, and contour match compared to the adjacent tissues, in terms of keloid formation, and in terms of patients' preference of the procedure. However, at least among the studies included herein, the overall patient satisfaction with the final outcome was not negatively affected by the adjunct of a CTG. These findings are well in agreement with short‐term results on aesthetic‐ and patient‐related outcomes following root coverage procedures recently summarised (Cairo et al., 2020).

Based on a primarily descriptive summary presented herein, a few recommendations for future studies and for the clinical praxis can be provided:

- RCT reporting on long‐term outcomes of single and multiple Miller class III/IV (RT 2 and 3) GR are not available so far, and therefore warranted.
- A higher number of long‐term assessments of other flap designs (e.g., TUN) and/or adjuncts (e.g., EMD, CM) in comparison to the ‘gold standard’ CAF + CTG are required for all type of GR.
- Evaluation of the aesthetic outcome and PROMs should be included, as these might be – next to the higher morbidity due to the second surgical site – relative shortcomings of the ‘gold standard’ CAF + CTG.
- Details on CTG harvesting (i.e., region, graft type/harvesting technique, graft thickness, positioning in relation to the flap) should be either reported or direct comparisons be performed to be able to evaluate whether a specific graft type/technique is more likely to achieve ‘creeping attachment’ and/or more favourable aesthetic outcomes.
- Individual patient data and frequency distributions instead of primarily mean values would allow a better understanding, whether any loss in mean root coverage is due to clustering to a few patients with a severe relapse or due to all patients losing slightly.

## 5 CONCLUSIONS

- For single Miller class I/II GR, CAF + CTG appears as the ‘gold standard’ also on the long‐term (i.e., ≥ 5 years of follow‐up), providing low residual RD (i.e., ≤ 0.5 mm) corresponding to a high RC rate >80%, and CRC was achieved in at least 2/3 of the patients.
- Other interventions (e.g., CAF + EMD, CAF + CM, CAF + grafting) were tested too seldomly to draw firm conclusions for the treatment of single GR; in the direct comparisons of the individual studies, CAF + CTG appeared as the advantageous technique.
- For multiple Miller class I‐III GR, CAF + CTG appears also as the best technique, providing a low mean residual RD with the potential for ‘creeping attachment’; ADMA as adjunct tended to have a higher relapse rate compared to CAF + CTG.
- For single or multiple Miller Class III/IV GR, no information on long‐term outcome is available.
- In general, comparing the mean residual RD values after 6 or 12 months to the final outcome, CAF + CTG was the only intervention for which a ‘creeping attachment’ became apparent.
- As CAF was the prevailing flap design (i.e., in 86.5% of the groups), no conclusion on other flap designs (e.g., TUN, LPF, etc.) can be drawn.
- Although CAF + CTG can be considered as the ‘gold standard’ in terms of clinical parameters, there might be some shortcomings in terms of tissue integration and aesthetic appearance.

## CONFLICT OF INTEREST

The authors declare there is no conflict of interest.

## Supporting information


**Appendix 1.** Prisma 2009 checklist.

**Appendix 2.** Details on the statistical analysis (including reference list).

**Appendix 3**. A panel of network plots for the secondary outcome parameters. The nodes refer to the interventions and the lines that link the nodes indicate the observed comparisons. The size of the nodes is proportional to the number of comparisons that include the node. The thickness of the lines is proportional to the number of trials that investigate the corresponding comparison.

**Appendix 4.** Flowchart of the inclusion process of studies for the systematic review.

**Appendix 5.** Reasons for exclusion of 32 full‐texts.

**Appendix 6.** Reporting frequency of potential predictors on the outcome in the individual studies in relation to the gingival recession type.

**Appendix 7.** Exemplarily references of studies investigating potential predictors on the outcome and stability of root coverage procedures.

**Appendix 8.** A panel of forest plots for all observed comparisons for each secondary outcome parameter. The unique observed comparisons and the included trials appear on the right and the left of the panel, respectively. The trials have been ordered chronologically. The x‐axis refers to the mean difference. The design of the trial (parallel group vs. split‐mouth design), the level of RoB (some concerns vs. high), and the smoking status of the participants (mixed vs. non‐smoker) are indicated with different line types (solid vs. dashed), colours (orange vs. red), and point shapes (circle vs. triangle), respectively. The vertical grey line above zero implies no difference between the compared interventions. A positive mean difference indicates that the second intervention in the comparison is more favorable. A forest plot for CRC stability is not included due to lack of variance data in the original publications.

**Appendix 9.** Bubble plots that illustrate the relationship between the observational period in months (x‐axis) and the relative treatment effect observed in each trial (y‐axis) for each secondary outcome parameter. Each point refers to a trial. The size of each point has been weighted by the inverse of the variance of the corresponding trial: the smaller the trial, the smaller the size of the point and vice‐versa. Different colours refer to the comparisons investigated in each trial. A positive mean difference indicates that the second intervention in the comparison is more favorable. A bubble plot for CRC stability is not included due to lack of variance data in the original publications.

**Appendix 10.** RoB assessment of the studies eligible for network meta‐analysis applying the Cochrane Collaboration's Tool for assessing RoB Version 2; green represents a low risk, yellow some concerns, and red a high risk.

## Data Availability

Data available upon reasonable request
