# Supplementary material for: Root coverage stability: A systematic overview of controlled clinical trials with at least 5 years of follow‐up
Source: Clin Exp Dent Res. 2021 Feb 9;7(5):692–710. doi: 10.1002/cre2.395 (PMC8543486; doi:10.1002/cre2.395)
Supplement: Supplementary file 2 — Appendix 2. Details on the statistical analysis (including reference list). [file CRE2-7-692-s006.docx]

**Appendix 2.** Details on the statistical analysis (including reference list).

All outcomes were measured using the mean difference, except for CRC, which was measured using the odds ratio in the logarithmic scale (log OR). For the trials with standard parallel group design, the variance of the mean difference was estimated as (Borenstein et al. 2009),

$$V_{MD}=\frac{v_{T}}{n_{T}}+\frac{v_{C}}{n_{C}}$$

where $v_{T}$ and $v_{C}$ are the variance in the treatment and control arm, and $n_{T}$ and $n_{C}$ are the size of the respective arms at the final point of the trial. The variance of log OR was estimated using the delta method as follows:

$$V_{LOR}=\frac{1}{{n_{T}p}_{T}\left( 1-p_{T} \right)}+\frac{1}{{n_{C}p}_{C}\left( 1-p_{C} \right)}$$

where $p_{T}$ and $p_{C}$ are CRC in the treatment and control arm. The common continuity correction of 0.5 was not required, since there was no zero cell(s) in any of the trials.

For the trials with a split-mouth design, an intra-patient correlation of 0.5 was considered to obtain a statistical correct within-trial variance for mean difference and log OR. Note that assuming a larger (or lower) positive intra-patient correlation will reduce (or increase) the variance around the effect measure further, and by extent, it will affect the conclusions concerning statistical significance. Then, the within-trial variance for mean difference is calculated using the following formula (Borenstein et al. 2009):

$$V_{MD}=\frac{v_{T}+v_{C}-2\rho\sqrt{v_{T}v_{C}}}{n}$$

where $\rho$ is the intra-patient correlation and $n$ is the number of ‘pairs’. In the case of log OR, the corresponding formula for the within-trial variance is

$$V_{LOR}=v_{T}+v_{C}-2\rho\sqrt{v_{T}v_{C}}$$

where $v_{T}$ and $v_{C}$ refer to the variance of the log odds in the treatment and control arm, and they are estimated using the delta method as follows:

$$v_{k}=\frac{1}{{n_{k}p}_{k}\left( 1-p_{k} \right)}, k=C,T$$

with $n_{C}=n_{T}=n$.

For each outcome, a Bayesian random-effects NMA (Dias et al. 2013) was planned, with the incorporation of consistency equations (Lu & Ades 2006) to estimate the internally coherent relative treatment effects for all possible comparisons (Caldwell 2014), and to provide an intervention hierarchy (i.e., from the best to the worst intervention) (Salanti et al. 2011). Additionally, the presence of possible moderators that may direct the recommendations to distinct patient sub-populations was planned to be investigated. For that purpose, the smoking status of the participants, the trial design (i.e., parallel group or split-mouth design), the observation period of the trial (in months), and the quality of the trials determined by the overall RoB level (i.e., low risk, some concerns, or high risk) were considered as important effect modifiers.

In this context, NMA requires that the network of interventions is connected; namely, each intervention is compared with at least another intervention. Furthermore, the analyst must defend the transitivity assumption regarding ‘joint randomization of interventions’ and the similarity of the included trials across the observed comparisons (Chaimani et al. 2017, Salanti 2012). Joint randomization implies that if there was a multi-arm trial with all interventions of interest, then it could be assumed that every patient would be randomized to any of these interventions. Nevertheless, in the present study, the included trials cannot be perceived as transitive across the observed comparisons concerning the characteristics mentioned above. In practice, moderator analysis is performed to adjust the relative treatment effects for intransitivity, provided that there is a sufficient number of trials in the observed comparisons to allow for enough power in the estimation of the regression coefficients and their standard error (Cooper et al. 2009, Jansen & Naci 2013). In the present study, moderator analysis was not possible in any of the outcomes due to the limited number of trials in the networks. In all networks, all observed comparisons were informed by only one trial, except for one comparison that included two trials (CAF vs. CAF + CTG) (Figure 1, Appendix 3). Consequently, it was not possible to perform NMA for any of the outcomes. Pairwise meta-analysis for the comparison ‘CAF vs. CAF + CTG’ was also not plausible due to considerable clinical and methodological heterogeneity in the included trials.

The results of the included trials for all outcomes and observed comparisons were presented by using a panel of forest plots, while taking into account the patient and trial characteristics mentioned above. Additionally, bubble plots were used to visually examine the association between the within-trial relative treatment effect and the observational period of the trials.

All analyses were performed in the statistical software R (version 4.0.2) (R Core Team 2020). The R-package ggplot2 (Wickham 2009) were used to draw the panel of forestplots and bubble plots, and the R-package pcnetmeta (Lin et al. 2017) to create the network plots.

**References**

Borenstein, M., L.V. Hedges, J.P.T. Higgins & H.R. Rothstein. 2009. Chapter 4: Effect Sizes Based on Means. In *Introduction to Meta-Analysis*, eds. John Wiley & Sons, Ltd., p.

Caldwell, D.M. (2014) An overview of conducting systematic reviews with network meta-analysis. *Syst Rev* **3,** 109.

Chaimani, A., D.M. Caldwell, T. Li, J.P.T. Higgins & G. Salanti (2017) Additional considerations are required when preparing a protocol for a systematic review with multiple interventions. *J Clin Epidemiol* **83,** 65-74.

Cooper, N.J., A.J. Sutton, D. Morris, A.E. Ades & N.J. Welton (2009) Addressing between-study heterogeneity and inconsistency in mixed treatment comparisons: Application to stroke prevention treatments in individuals with non-rheumatic atrial fibrillation. *Stat Med* **28,** 1861-1881.

Dias, S., A.J. Sutton, A.E. Ades & N.J. Welton (2013) Evidence synthesis for decision making 2: a generalized linear modeling framework for pairwise and network meta-analysis of randomized controlled trials. *Med Decis Making* **33,** 607-617.

Jansen, J.P. & H. Naci (2013) Is network meta-analysis as valid as standard pairwise meta-analysis? It all depends on the distribution of effect modifiers. *BMC Med* **11,** 159.

Lin, L., J. Zhang, J.S. Hodges & H. Chu (2017) Performing arm-based networkmeta-analysis in R with the pcnetmeta package. *J Stat Softw* **80,**

Lu, G. & A.E. Ades (2006) Assessing evidence inconsistency in mixed treatment comparisons. *J Am Stat Assoc* **101,** 447-459.

R Core Team (2020) R: A Language and Environment for Staistical Computing.

Salanti, G. (2012) Indirect and mixed-treatment comparison, network, or multiple-treatments meta-analysis: many names, many benefits, many concerns for the next generation evidence synthesis tool. *Res Synth Methods* **3,** 80-97.

Salanti, G., A.E. Ades & J.P. Ioannidis (2011) Graphical methods and numerical summaries for presenting results from multiple-treatment meta-analysis: an overview and tutorial. *J Clin Epidemiol* **64,** 163-171.

Wickham, H. 2009. *ggplot2: Elegant Graphics for Data Analysis.* Springer-Verlag New York,
