# Supplementary figures and images for: Root coverage stability: A systematic overview of controlled clinical trials with at least 5 years of follow‐up
Source: Clin Exp Dent Res. 2021 Feb 9;7(5):692–710. doi: 10.1002/cre2.395 (PMC8543486; doi:10.1002/cre2.395)

**RD reduction**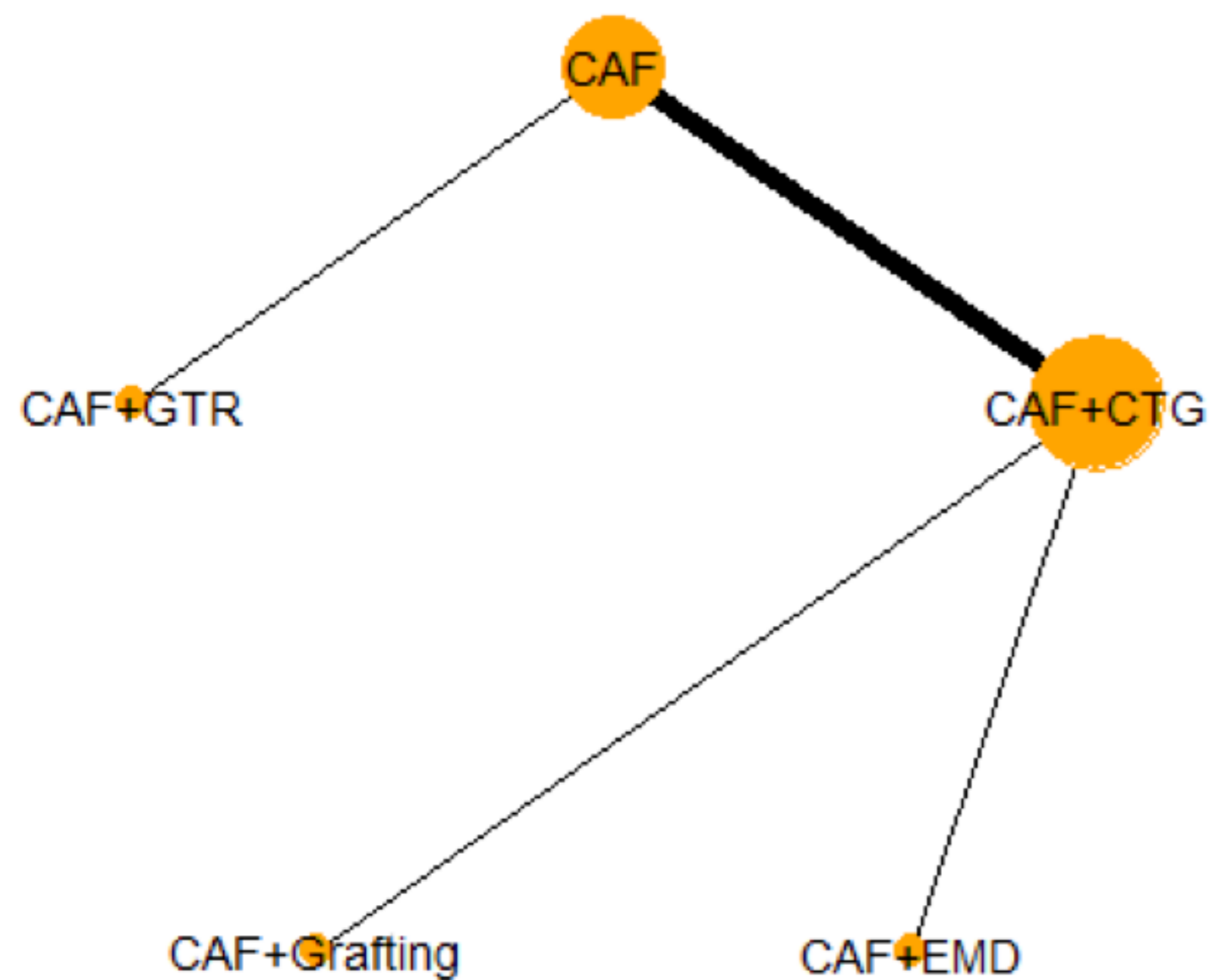**RD stability**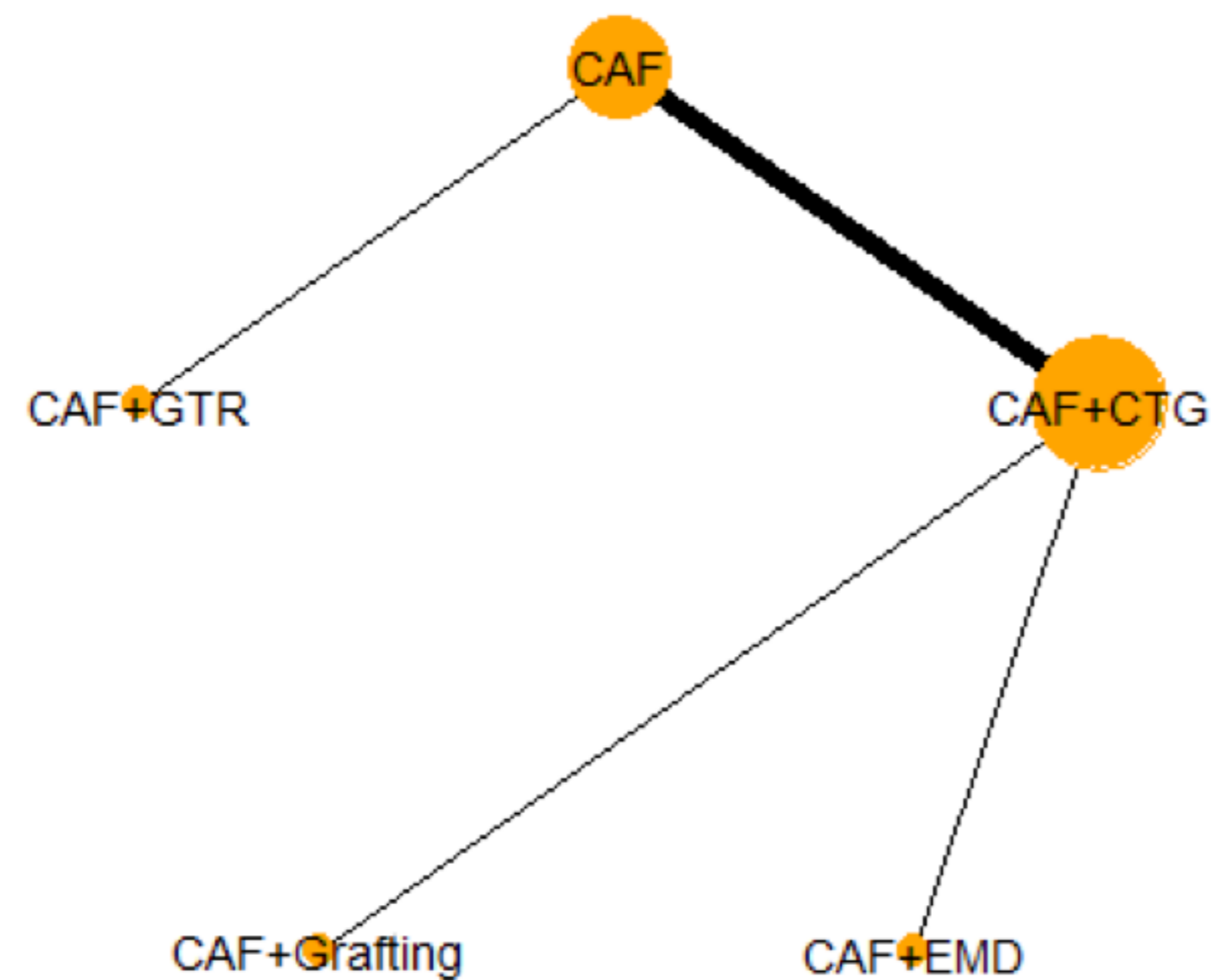**RC**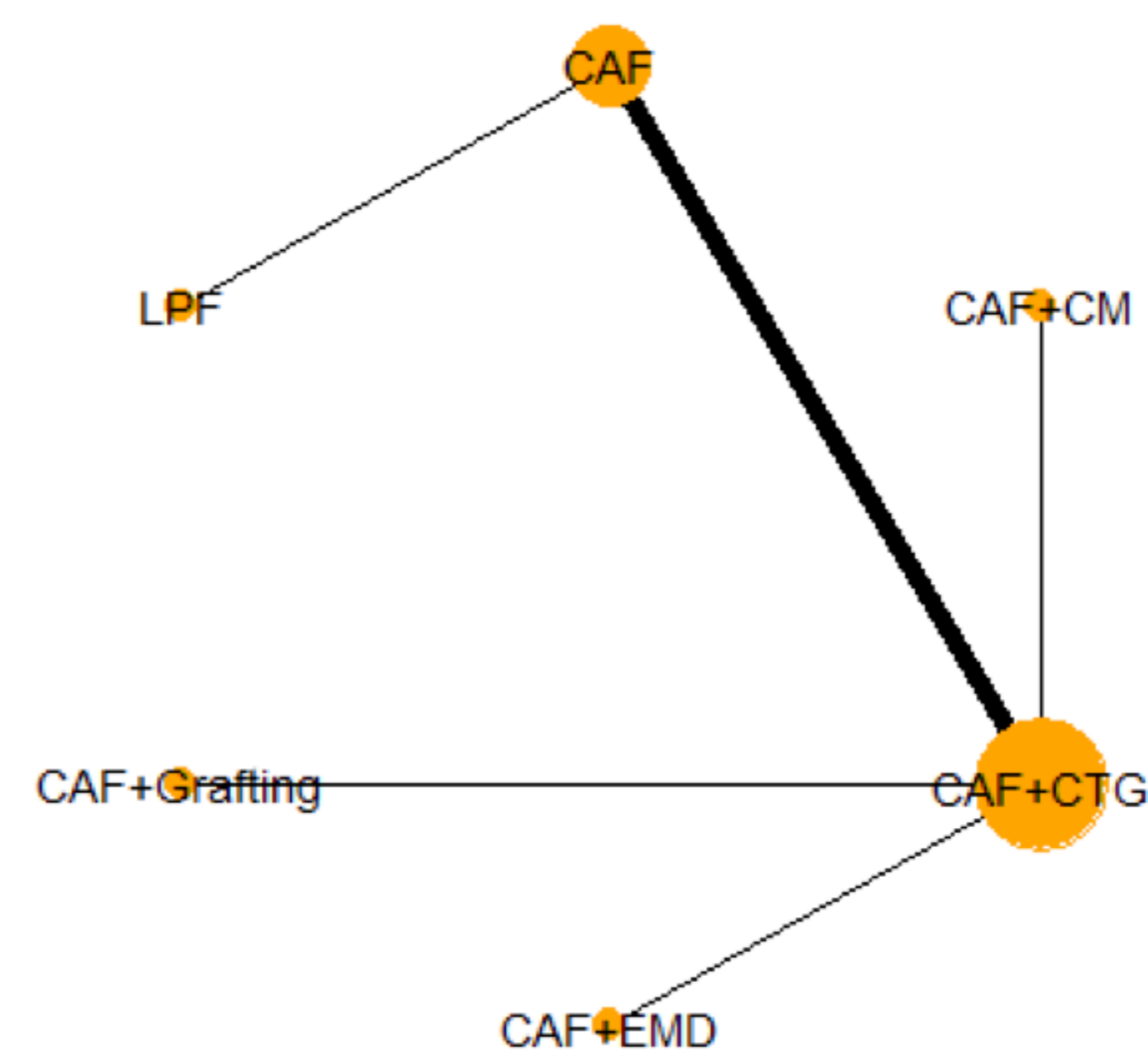**RC stability**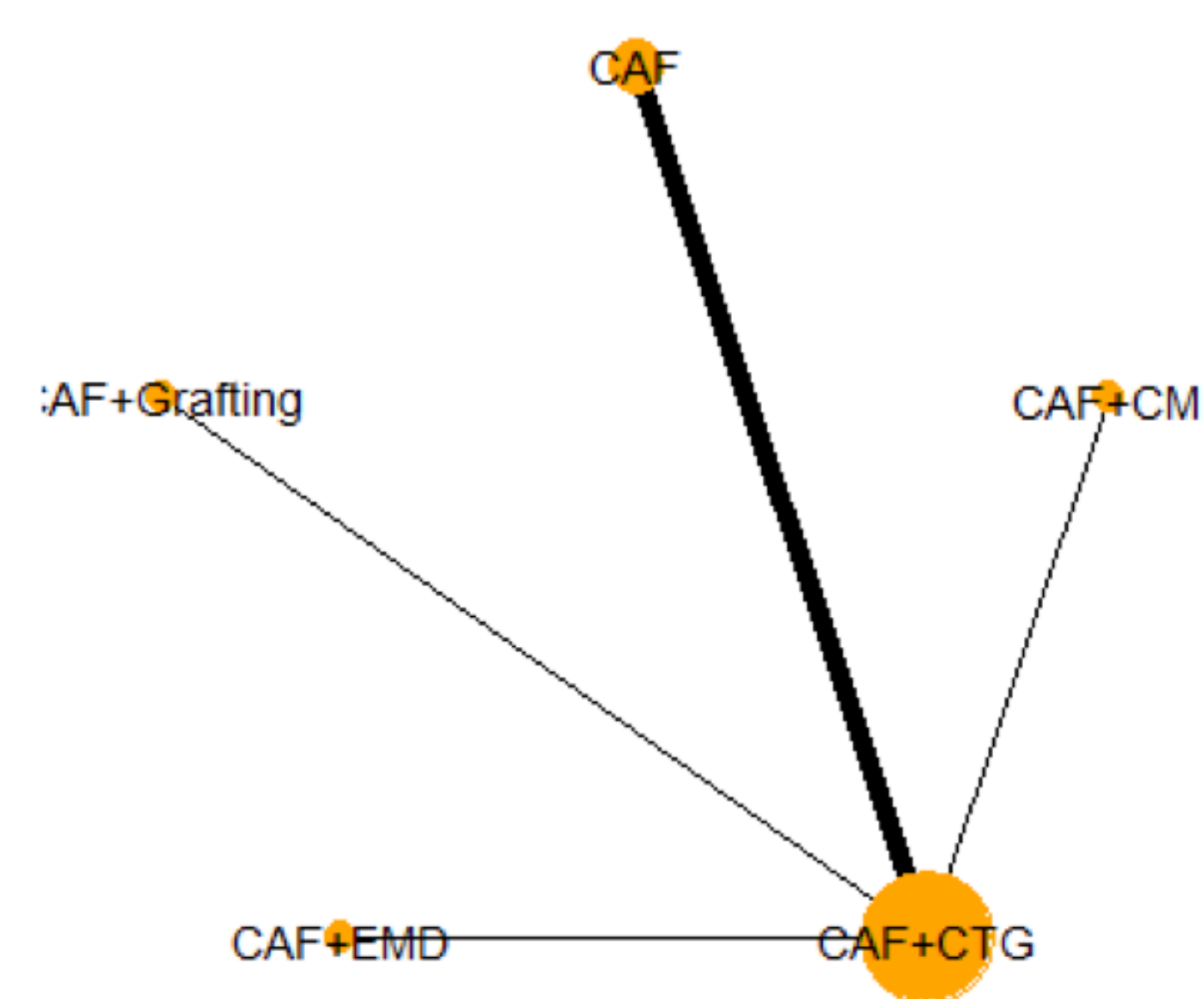**KTW**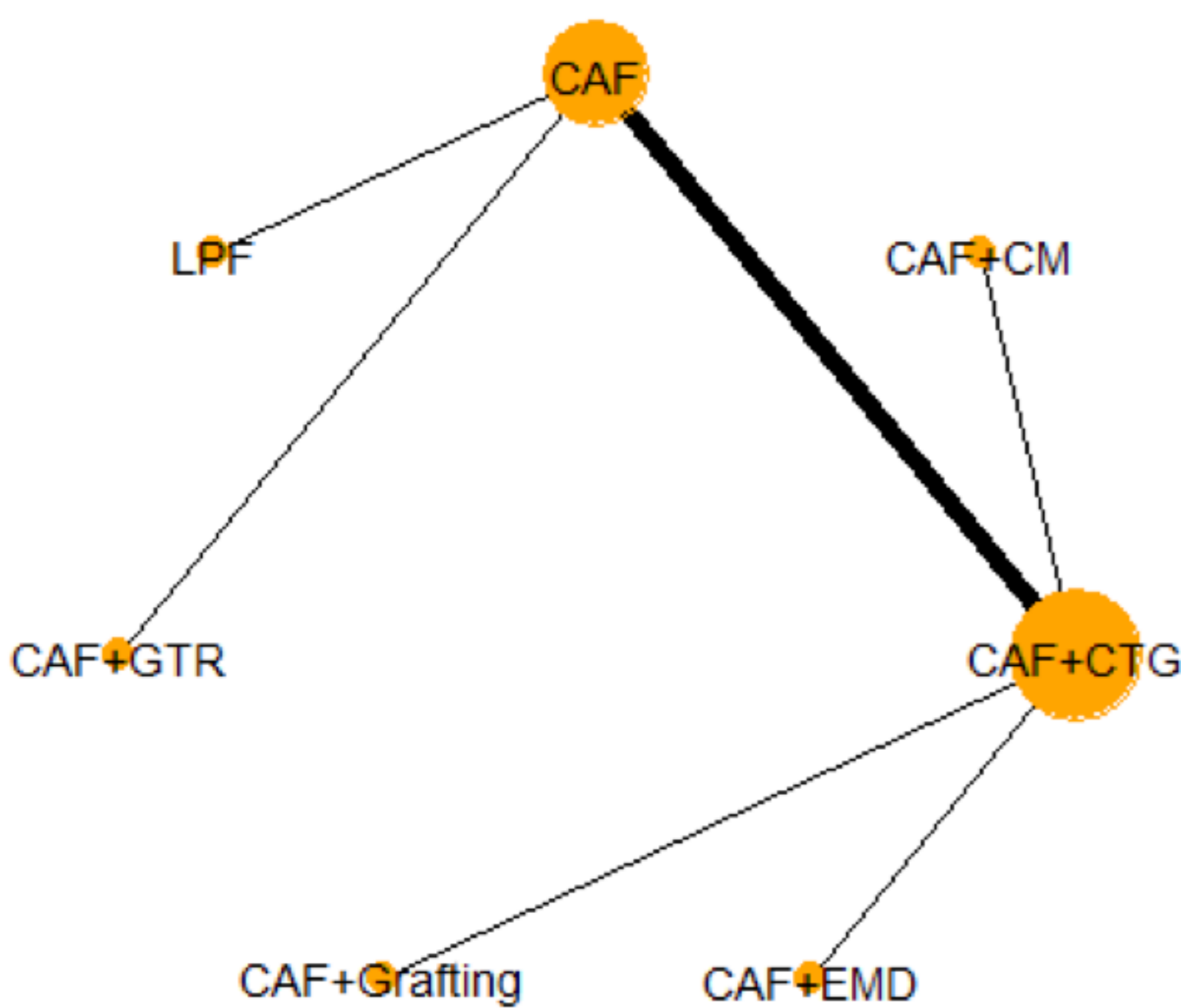**KTW increase**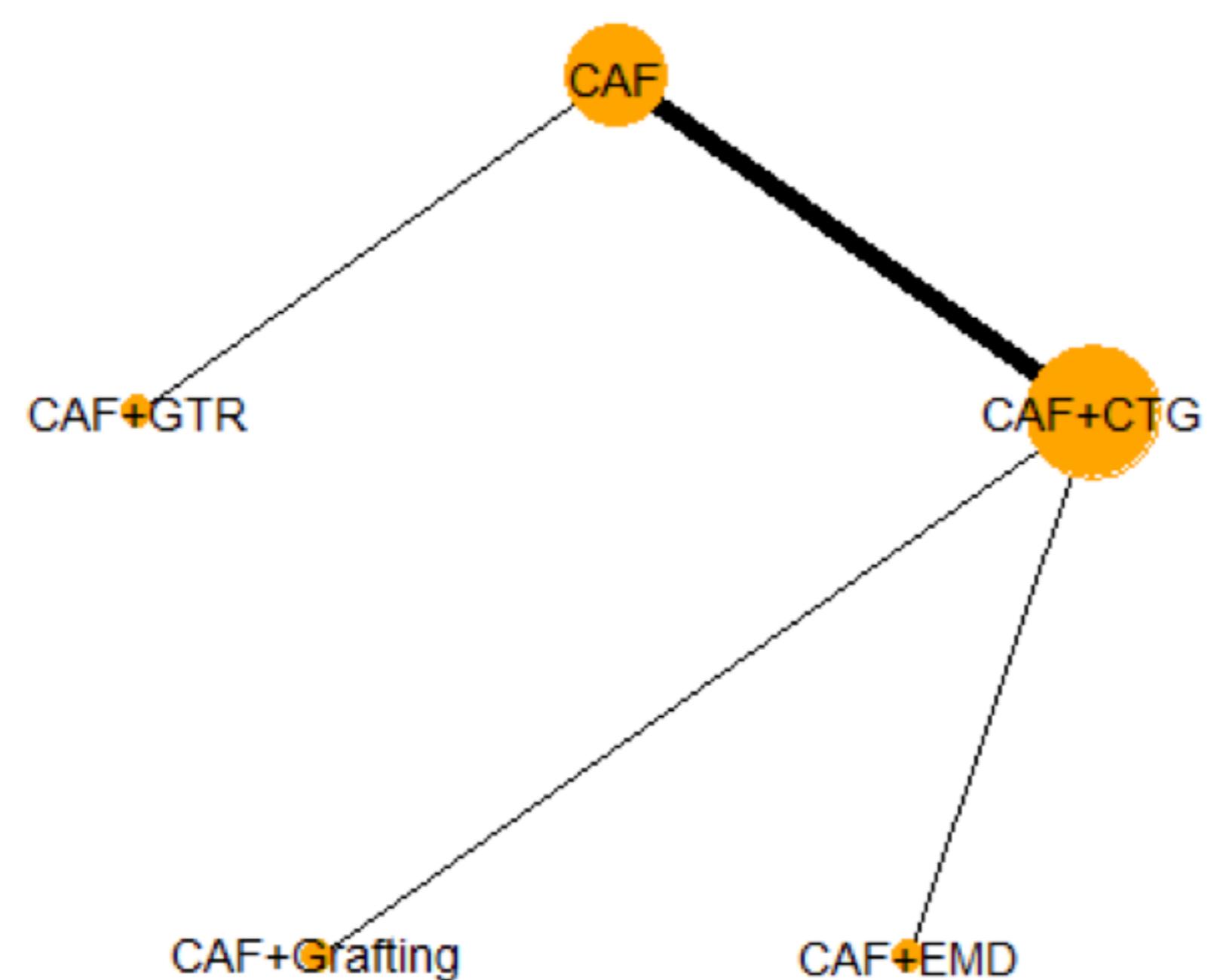**KTW stability**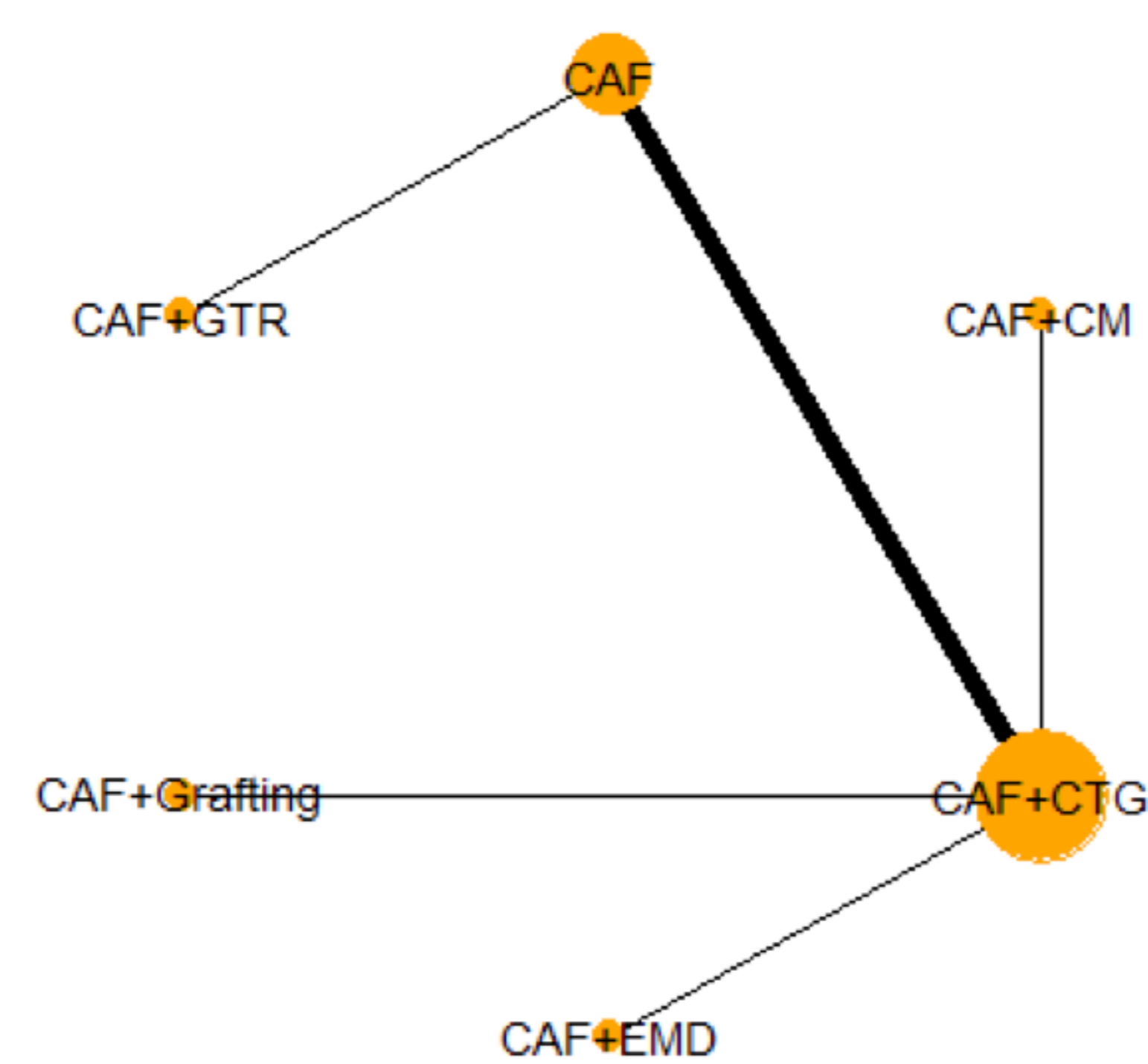

Supplement: Supplementary file 3 — Appendix 3. A panel of network plots for the secondary outcome parameters. The nodes refer to the interventions and the lines that link the nodes indicate the observed comparisons. The size of the nodes is proportional to the number of comparisons that include the node. The thickness of the lines is proportional to the number of trials that investigate the corresponding comparison. [file CRE2-7-692-s007.pdf]

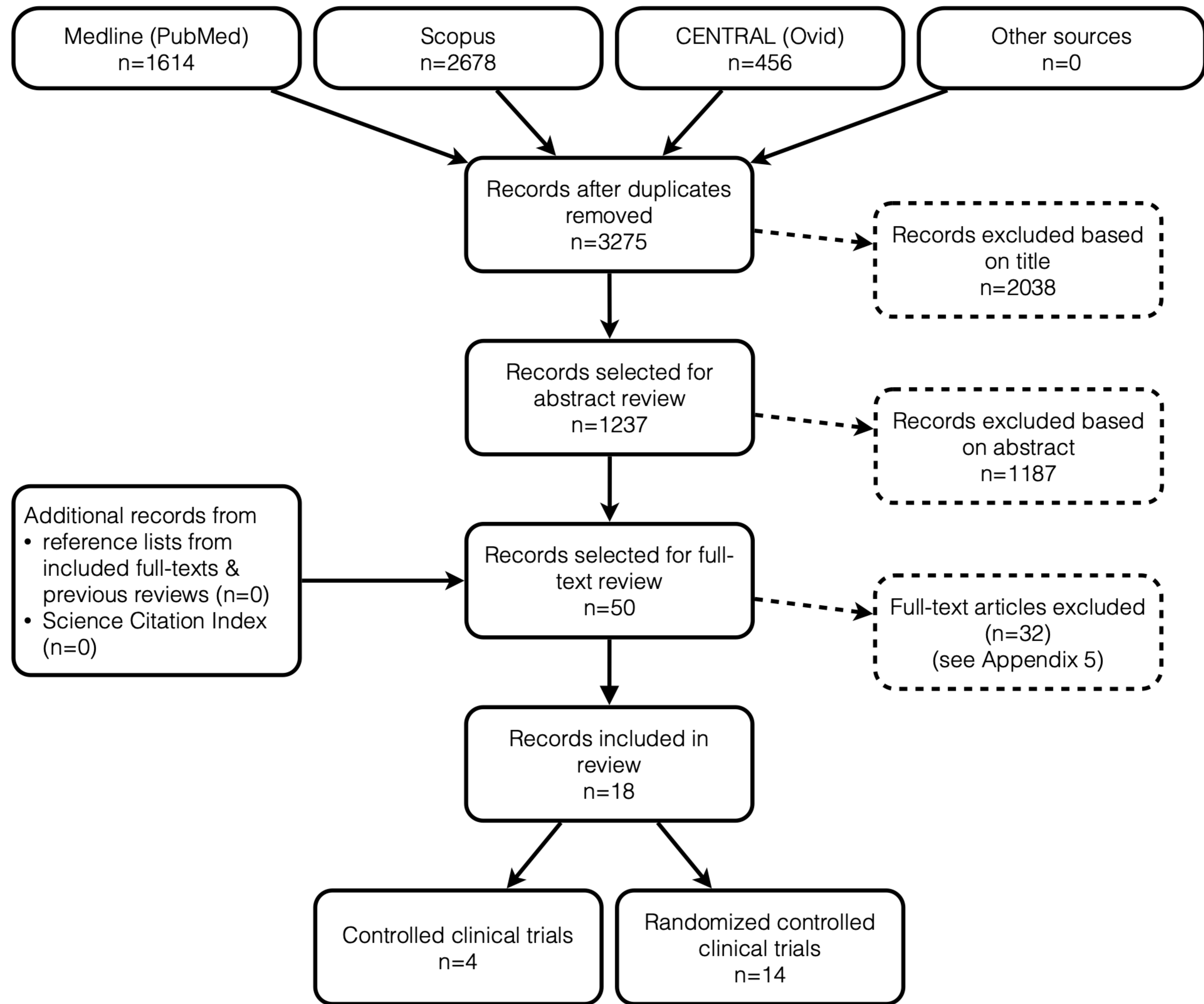

Supplement: Supplementary file 4 — Appendix 4. Flowchart of the inclusion process of studies for the systematic review. [file CRE2-7-692-s009.pdf]

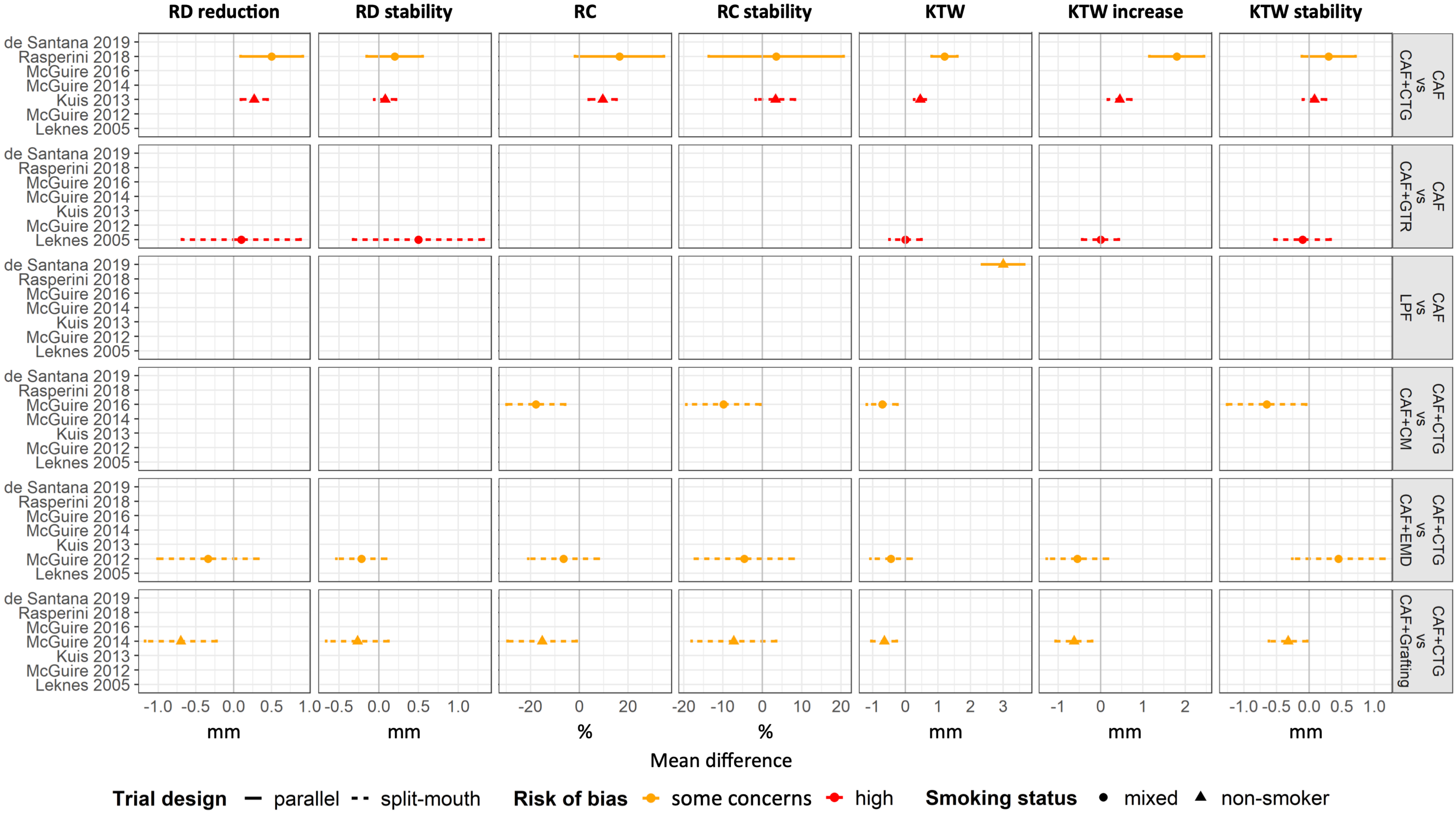

Supplement: Supplementary file 8 — Appendix 8. A panel of forest plots for all observed comparisons for each secondary outcome parameter. The unique observed comparisons and the included trials appear on the right and the left of the panel, respectively. The trials have been ordered chronologically. The x‐axis refers to the mean difference. The design of the trial (parallel group vs. split‐mouth design), the level of RoB (some concerns vs. high), and the smoking status of the participants (mixed vs. non‐smoker) are indicated with different line types (solid vs. dashed), colours (orange vs. red), and point shapes (circle vs. triangle), respectively. The vertical grey line above zero implies no difference between the compared interventions. A positive mean difference indicates that the second intervention in the comparison is more favorable. A forest plot for CRC stability is not included due to lack of variance data in the original publications. [file CRE2-7-692-s001.pdf]
