# Supplementary material for: Root coverage stability: A systematic overview of controlled clinical trials with at least 5 years of follow‐up
Source: Clin Exp Dent Res. 2021 Feb 9;7(5):692–710. doi: 10.1002/cre2.395 (PMC8543486; doi:10.1002/cre2.395)
Supplement: Supplementary file 5 — Appendix 5. Reasons for exclusion of 32 full‐texts. [file CRE2-7-692-s002.docx]

**Appendix 5.** Reasons for exclusion of 32 full-texts (including reference list).

| **Study (year)** | **Reason for exclusion** |
| --- | --- |
| Agudio et al. (2016) | Augmentation of keratinized tissue without root coverage |
| Atanassov (2001) | No control group |
| Baghele & Pol (2012) | < 5 years follow-up |
| Bruno (1994) | Description of a technique |
| Bruno (1999) | Description of a technique |
| Caffesse & Espinel (1981) | < 5 years follow-up |
| Caffesse et al. (1987) | < 5 years follow-up |
| Cardaropoli et al. (2014) | < 5 years follow-up |
| Carvalho et al. (1982) | Description of a technique |
| Cortellini et al. (2009) | < 5 years follow-up |
| Daniel & Cheru (1990) | < 5 years follow-up |
| Espinel & Caffesse (1981a) | < 5 years follow-up |
| Espinel & Caffesse (1981b) | < 5 years follow-up |
| Esteibar et al. (2011) | < 5 years follow-up |
| Harris (2000) | < 5 years follow-up |
| Harris et al. (2007) | < 5 years follow-up |
| Harris et al. (2005) | < 5 years follow-up |
| Jaiswal et al. (2012) | < 5 years follow-up |
| Jovicic et al. (2013) | < 5 years follow-up |
| Khatiblou (1982) | Report of 2 cases |
| Mahajan et al. (2012) | < 5 years follow-up |
| Nickles et al. (2010) | Longer follow-up included (Petsos et al. 2020) |
| Pietruska et al. (2019) | < 5 years follow-up |
| Pollack (1990) | Description of a technique |
| Ricci et al. (1996) | < 5 years follow-up |
| Ross et al. (1986) | Description of a technique |
| Santos et al. (2017) | Report of a single case |
| Sumana et al. (2017) | < 5 years follow-up |
| Waite (1984) | < 5 years follow-up |
| Yuce et al. (2015) | < 5 years follow-up |
| Zanwar et al. (2014) | < 5 years follow-up |
| Zucchelli et al. (2010) | < 5 years follow-up |

**References excluded studies**

Agudio, G., P. Cortellini, J. Buti & G. Pini Prato (2016) Periodontal Conditions of Sites Treated With Gingival Augmentation Surgery Compared With Untreated Contralateral Homologous Sites: An 18- to 35-Year Long-Term Study. J Periodontol 87, 1371-1378.

Atanassov, D.T. (2001) Long term results in the treatment of multiple gingival recessions. Folia Med (Plovdiv) 43, 136-139.

Baghele, O.N. & D.G. Pol (2012) An evaluation of the effectiveness and predictability of transpositional flap vs connective tissue graft for coverage of Miller’s class-I and class-II facial marginal tissue recession lesions: a clinical study. Indian J Dent Res 23, 195-202.

Bruno, J.F. (1994) Connective tissue graft technique assuring wide root coverage. Int J Periodontics Restorative Dent 14, 126-137.

Bruno, J.F. (1999) A subepithelial connective tissue graft procedure for optimum root coverage. Atlas Oral Maxillofac Surg Clin North Am 7, 11-28.

Caffesse, R.G., S.R. Alspach, E.C. Morrison & F.G. Burgett (1987) Lateral sliding flaps with and without citric acid. Int J Periodontics Restorative Dent 7, 42-57.

Caffesse, R.G. & M.C. Espinel (1981) Lateral sliding flap with a free gingival graft technique in the treatment of localized gingival recessions. Int J Periodontics Restorative Dent 1, 22-29.

Cardaropoli, D., L. Tamagnone, A. Roffredo & L. Gaveglio (2014) Coronally advanced flap with and without a xenogenic collagen matrix in the treatment of multiple recessions: a randomized controlled clinical study. Int J Periodontics Restorative Dent 34 Suppl 3, s97-102.

Carvalho, J.C., F.E. Pustiglioni & S. Kon (1982) Combination of a connective tissue pedicle flap with a free gingival graft to cover localized gingival recession. Int J Periodontics Restorative Dent 2, 26-33.

Cortellini, P., M. Tonetti, C. Baldi, L. Francetti, G. Rasperini, R. Rotundo, M. Nieri, D. Franceschi, A. Labriola & G.P. Prato (2009) Does placement of a connective tissue graft improve the outcomes of coronally advanced flap for coverage of single gingival recessions in upper anterior teeth? A multi-centre, randomized, double-blind, clinical trial. J Clin Periodontol 36, 68-79.

Daniel, A. & R. Cheru (1990) Treatment of localised gingival recession with subpedicle connective tissue graft and free gingival auto graft--a comparative clinical evaluation. J Indian Dent Assoc 61, 294-297.

Espinel, M.C. & R.G. Caffesse (1981a) Comparison of the results obtained with the laterally positioned pedicle sliding flap-revised technique and the lateral sliding flap with a free gingival graft technique in the treatment of localized gingival recessions. Int J Periodontics Restorative Dent 1, 30-37.

Espinel, M.C. & R.G. Caffesse (1981b) Lateral positioned pedicle sliding flap-revised technique in the treatment of localized gingival recessions. Int J Periodontics Restorative Dent 1, 42-51.

Esteibar, J.R., L.A. Zorzano, E.E. Cundín, J.D. Blanco & J.R. Medina (2011) Complete root coverage of Miller Class III recessions. Int J Periodontics Restorative Dent 31, e1-7.

Harris, R.J. (2000) A comparative study of root coverage obtained with an acellular dermal matrix versus a connective tissue graft: results of 107 recession defects in 50 consecutively treated patients. Int J Periodontics Restorative Dent 20, 51-59.

Harris, R.J., L.E. Harris, C.R. Harris & A.J. Harris (2007) Evaluation of root coverage with two connective tissue grafts obtained from the same location. Int J Periodontics Restorative Dent 27, 333-339.

Harris, R.J., L.H. Miller, C.R. Harris & R.J. Miller (2005) A comparison of three techniques to obtain root coverage on mandibular incisors. J Periodontol 76, 1758-1767.

Jaiswal, G.R., R. Kumar, P.M. Khatri, S.G. Jaiswal & M.L. Bhongade (2012) The effectiveness of enamel matrix protein (Emdogain(®)) in combination with coronally advanced flap in the treatment of multiple marginal tissue recession: A clinical study. J Indian Soc Periodontol 16, 224-230.

Jovicić, B., Z. Lazić, M. Nedić, S. Matijević & A. Gostović-Spadijer (2013) Therapeutic efficacy of connective tissue autotransplants with periosteum and platelet rich plasma in the menagement of gingival recession. Vojnosanit Pregl 70, 664-669.

Khatiblou, F.A. (1982) Combined use of the lateral sliding flap and free gingival graft procedures in treatment of gingival recession. Periodontal Case Rep 4, 5-8.

Mahajan, A., A. Bharadwaj & P. Mahajan (2012) Comparison of periosteal pedicle graft and subepithelial connective tissue graft for the treatment of gingival recession defects. Aust Dent J 57, 51-57.

Nickles, K., P. Ratka-Krüger, E. Neukranz, P. Raetzke & P. Eickholz (2010) Ten-year results after connective tissue grafts and guided tissue regeneration for root coverage. J Periodontol 81, 827-836.

Pietruska, M., A. Skurska, Ł. Podlewski, R. Milewski & J. Pietruski (2019) Clinical evaluation of Miller class I and II recessions treatment with the use of modified coronally advanced tunnel technique with either collagen matrix or subepithelial connective tissue graft: A randomized clinical study. J Clin Periodontol 46, 86-95.

Pollack, R.P. (1990) Root coverage using submarginal free mucosal grafts in conjunction with a retained gingival collar. Compendium 11, 160, 162-4.

Ricci, G., M. Silvestri, G. Rasperini & V. Cattaneo (1996) Root coverage: a clinical/statistical comparison between subpedicle connective tissue graft and laterally positioned full thickness flaps. J Esthet Dent 8, 66-73.

Ross, S.E., H.W. Crosetti, A. Gargiulo & D.W. Cohen (1986) The double papillae repositioned flap--an alternative. I. Fourteen years in retrospect. Int J Periodontics Restorative Dent 6, 46-59.

Santos, F.R., C.L. Storrer, E.J. Cunha, L.M. Ulbrich, C.A. Lopez & T.M. Deliberador (2017) Comparison of conventional and semilunar coronally positioned flap techniques for root coverage in teeth with cervical abrasion restored with pink resin. Clin Cosmet Investig Dent 9, 7-11.

Sumana, S.Z., S.L.C. Masulili & R. Lessang (2017) Root coverage using the subepithelial connective tissue graft or the acellular dermal matrix for the treatment of gingival recession: A clinical study. International Journal of Applied Pharmaceutics 9,

Waite, I.M. (1984) An assessment of the postsurgical results following the combined laterally positioned flap and gingival graft procedure. Quintessence Int Dent Dig 15, 441-450.

Yuce, H.B., H.A. Turkal, O. Karatas & S.S. Altintepe (2015) Root esthetic score (RES) comparisons of FGG and PE-FGG procedures in Miller class II and III recessions in mandibular anterior teeth. Cumhuriyet Dental Journal 18,

Zanwar, K., M. Laxmanrao Bhongade, K. Kumar Ganji, S. B Koudale & P. Gowda (2014) Comparative evaluation of efficacy of stem cells in combination with PLA/PGA membrane versus sub-epithelial connective tissue for the treatment of multiple gingival recession defects: a clinical study. J Stem Cells 9, 253-267.

Zucchelli, G., M. Mele, M. Stefanini, C. Mazzotti, M. Marzadori, L. Montebugnoli & M. de Sanctis (2010) Patient morbidity and root coverage outcome after subepithelial connective tissue and de-epithelialized grafts: a comparative randomized-controlled clinical trial. J Clin Periodontol 37, 728-738.
