# Supplementary material for: Root coverage stability: A systematic overview of controlled clinical trials with at least 5 years of follow‐up
Source: Clin Exp Dent Res. 2021 Feb 9;7(5):692–710. doi: 10.1002/cre2.395 (PMC8543486; doi:10.1002/cre2.395)
Supplement: Supplementary file 6 — Appendix 6. Reporting frequency of potential predictors on the outcome in the individual studies in relation to the gingival recession type. [file CRE2-7-692-s008.docx]

**Appendix 6.** Reporting frequency of potential predictors on the outcome in the individual studies in relation to the gingival recession type.

| **Study (year)** | **Gingival phenotype/ thickness** | **Recession width** | **KTW** | **Flap details** | | **CTG details** | | | | **Root conditioning** | **Cervical lesion** | | | **Time-point suture removal** | **SPT performance & interval** | **Long-term surveillance of oral hygiene habits** |
| --- | --- | --- | --- | --- | --- | --- | --- | --- | --- | --- | --- | --- | --- | --- | --- | --- |
|  |  |  |  | **CAF incision design** | **Flap positioning in relation to the CEJ** | **Donor region** | **Harvesting technique** | **CTG thickness** | **Coverage by the flap** |  | **CEJ detectable** | **Step present** | **Step restored** |  |  |  |
| **Single gingival recessions with Miller class I/II** | | | | | | | | | | | | | | | | |
| Leknes et al. (2005) | only at FE | reported | reported | vertical releasing incisions | 1-2 mm above the CEJ | not relevant (no CTG) | | | | polishing | NR | NR | NR | NR | 1x/year | regular re-instructions |
| Pini-Prato et al. (2011) | NR | NR | reported | vertical releasing incisions | at the level or above the CEJ | not relevant (no CTG) | | | | polishing vs. root planing | all identifiable | absence of grooves, irregularities, caries | NR | 10 days | 2x/year | regular re-instructions |
| McGuire et al. (2012) | NR | NR | reported | vertical releasing incisions | at the level of the CEJ | palate | split-flap | NR | covered | root planing, recontouring & 24% EDTA | NR | NR | NR | NR | NR | NR |
| Kuis et al. (2013) | NR | NR | reported | vertical releasing incisions | covering the CEJ | palate | split-flap | NR | covered | NR | all identifiable | NR | NR | 14 days | 2x/year | NR |
| McGuire et al. (2014) | NR | NR | reported | vertical releasing incisions | at the level of the CEJ | palate | split-flap | NR | covered | root planing, recontouring & 24% EDTA | NR | NR | NR | NR | 2-4x/year at periodontist or GP | NR |
| McGuire & Scheyer (2016) | NR | reported but no data presented | reported | vertical releasing incisions | at the level or slightly above the CEJ | palate | NR | NR | covered | root planing, recontouring & 24% EDTA | NR | NR | NR | NR | 2-4x/year at periodontist or GP | NR |
| Rasperini et al. (2018) | NR | reported | reported | vertical releasing incisions | covering the CEJ | palate | NR | 1-2 mm | covered | root planing | all identifiable | ≤ 1 mm at the CEJ | NR | 7-9 days | 2-3x/year | regular re-instructions |
| Francetti et al. (2018) | NR | reported but no data presented | reported | vertical releasing incisions | CTG at the level of the CEJ, flap NR | palate | NR | 1-2 mm | NR | root planing/ debridement | NR | < 1 mm at the CEJ | NR | NR | NR | NR |
| de Santana et al. (2019) | NR | NR | reported | vertical releasing incisions | CAF: NR  LPF: 1 mm above the CEJ | not relevant (no CTG) | | | | root planing | NR | absence of caries | NR | 14 days | 2x/year | regular re-instructions |
| **Multiple gingival recessions with Miller class I/II** | | | | | | | | | | | | | | | | |
| Zucchelli et al. (2014) | NR | NR | reported | no vertical releasing incisions | above the CEJ | palate | deepithelialised | ≤ 1 mm | NR | root planing, recontouring & 24% EDTA | all identifiable | ≤ 1 mm at the CEJ | NR | 14 days | 4x/year | regular re-instructions |
| Kroiss et al. (2019) | reported | NR | reported | no vertical releasing incisions | 1 mm above the CEJ | palate | split-flap  no periosteum | NR | covered | root planing & recontouring | NR | NR | NR | 14 days | NR | regular re-instructions |
| Tavelli et al. (2019) | GT at BL, phenotype at FE | NR | reported | vertical releasing incisions | 2 mm above the CEJ | not relevant (no CTG) | | | | root planing, recontouring & 24% EDTA | - | no NCCL | - | 14 days | ≥ 2x/year | NR |
| **Multiple gingival recessions with Miller class I/II/III** | | | | | | | | | | | | | | | | |
| Pini-Prato et al. (2010) | NR | NR | NR | with & without vertical releasing incisions | above the CEJ | palate | split-flap | NR | covered | root planing | all identifiable | ≤ 1 mm at the CEJ | NR | 7 days | 2x/year | NR |
| **Single and multiple gingival recessions mixed with Miller class I/II** | | | | | | | | | | | | | | | | |
| Paolantonio et al. (1997) | NR | NR | reported | vertical releasing incisions | at the level of the CEJ | palate | split-flap | NR | Covered as much as possible | root planing | NR | NR | NR | 7-10 days | 4x/year | controlled throughout the study |
| Dominiak et al. (2006) | reported in 2 groups | reported | reported | NR | NR | NR | NR | NR | NR | root planing & recontouring | NR | NR | NR | NR | NR | NR |
| Moslemi et al. (2011) | NR | reported | reported | vertical releasing incisions | at the level of the CEJ | NR | NR | 2-3 mm | NR | root planing | NR | cervical caries excluded | NR | NR | routine visits at GP | assessment of the brushing technique at FE |
| Barootchi et al. (2019) | reported | NR | reported | no vertical releasing incisions | 1 mm above the CEJ | palate | split-flap  with periosteum | NR | CTG covered, CTG with epithelial collar exposed | root planing & recontouring | NR | NR | NR | 10-14 days | 2-3x/year | NR |
| Petsos et al. (2020) | NR | reported | reported | vertical releasing incisions | Envelope pouch + CTG: below CEJ  CAF + GTR: NR | palate | split-flap  no periosteum | 2 mm | exposed | root planing | all identifiable | NR | NR | 10 days | regular until 24 months, then not regular at GP | regular re-instructions only until 24 months |
| **Frequency of NR**  **[n (%)]** | **13/18**  **(72.2)** | **11/18 (61.1)** | **1/18**  **(5.6)** | **1/18**  **(5.6)** | **1/18**  **(5.6)** | **2/14**  **(14.3)** | **5/14**  **(35.7)** | **9/14**  **(64.3)** | **4/14**  **(28.6)** | **1/18**  **(5.6)** | **11/18**  **(61.1)** | **10/18**  **(55.6)** | **17/18**  **(94.4.)** | **7/18**  **(38.9)** | **4/18**  **(22.2)** | **9/18**  **(50.0)** |

*BL, baseline; CAF, coronally advanced flap; CEJ, cemento-enamel junction; CTG, connective tissue graft; EDTA, ethylenediaminetetraacetic acid; FE, final evaluation; GP, general practitioner; KTW, keratinized tissue width; NCCL, non-carious cervical lesion; NR, not reported; SPT, supportive periodontal treatment; TUN, tunnel technique.*
