# Supplementary material for: Root coverage stability: A systematic overview of controlled clinical trials with at least 5 years of follow‐up
Source: Clin Exp Dent Res. 2021 Feb 9;7(5):692–710. doi: 10.1002/cre2.395 (PMC8543486; doi:10.1002/cre2.395)
Supplement: Supplementary file 7 — Appendix 7. Exemplarily references of studies investigating potential predictors on the outcome and stability of root coverage procedures. [file CRE2-7-692-s004.docx]

**Appendix 7.** Exemplarily references of studies investigating potential predictors on the outcome and stability of root coverage procedures.

| **Parameter** | | **References** |
| --- | --- | --- |
| ***Gingival phenotype/thickness*** | | Baldi et al. 2019  Cairo et al. 2016  Cortellini & Bissada 2018  Huang et al. 2005  Hwang & Wang 2006  Rasperini et al. 2019  Rasperini et al. 2020  Tavelli et al. 2019b |
| ***Gingival recession width*** | | Chambrone et al. 2010 |
| ***KTW*** | | Barootchi et al. 2019  Cairo et al. 2017  Chambrone et al. 2019b  Pini-Prato et al. 2011  Pini-Prato et al. 2012  Pini-Prato et al. 2018b  Pini-Prato et al. 2018a  Pini-Prato et al. 2019  Tavelli et al. 2019a  Tavelli et al. 2019b |
| ***Flap details*** | |  |
|  | CAF incision design | Skurska et al. 2015  Zucchelli et al. 2019 |
|  | Flap positioning in relation to the CEJ | Cairo 2017  Chambrone et al. 2019b  Pini-Prato et al. 2005 |
| ***CTG details*** | |  |
|  | Donor region | Tavelli et al. 2019c |
|  | Harvesting technique | Chambrone & Tatakis 2015  Tavelli et al. 2019d |
|  | CTG thickness | Moisa et al. 2019 |
|  | Coverage by the flap | Dodge et al. 2018 |
| ***Root conditioning*** | | Barootchi et al. 2018  Chambrone & Tatakis 2015  Chambrone et al. 2019a  Chambrone et al. 2019b  Karam et al. 2016  Pini-Prato et al. 1999  Tatakis et al. 2015 |
| ***Cervical lesion*** | |  |
|  | CEJ detectable | Goldstein et al. 2002  Pini-Prato et al. 2018a  Pini-Prato et al. 2018b  Rasperini et al. 2018  Santamaria et al. 2018 |
|  | Step present |  |
|  | Step restorable |  |
| ***Time-point suture removal*** | | Tatakis & Chambrone 2016 |
| ***SPT performance & interval***  ***Long-term surveillance of oral hygiene habits*** | | Cairo et al. 2014  Dai et al. 2019  Leknes et al. 2005  McGuire et al. 2014  Moslemi et al. 2011  Pini-Prato et al. 2018a  Rasperini et al. 2018  Zucchelli & De Sanctis 2005  Zucchelli et al. 2014  Zucchelli et al. 2018 |

*CEJ, cemento-enamel junction; CTG, connective tissue graft; KTW, keratinized tissue width; SPT, supportive periodontal treatment.*

**References**

Baldi, C., G. Pini-Prato, U. Pagliaro, M. Nieri, D. Saletta, L. Muzzi & P. Cortellini (1999) Coronally advanced flap procedure for root coverage. Is flap thickness a relevant predictor to achieve root coverage? A 19-case series. *J Periodontol* 70**,** 1077-1084.

Barootchi, S., L. Tavelli, R. Di Gianfilippo, H.Y. Byun, T.J. Oh, L. Barbato, F. Cairo & H.L. Wang (2019) Long term assessment of root coverage stability using connective tissue graft with or without an epithelial collar for gingival recession treatment. A 12-year follow-up from a randomized clinical trial. *J Clin Periodontol*

Barootchi, S., L. Tavelli, A. Ravidà, C.W. Wang & H.L. Wang (2018) Effect of EDTA root conditioning on the outcome of coronally advanced flap with connective tissue graft: a systematic review and meta-analysis. *Clin Oral Investig* 22**,** 2727-2741.

Cairo, F. (2017) Periodontal plastic surgery of gingival recessions at single and multiple teeth. *Periodontol 2000* 75**,** 296-316.

Cairo, F., P. Cortellini, A. Pilloni, M. Nieri, S. Cincinelli, F. Amunni, G. Pagavino & M.S. Tonetti (2016) Clinical efficacy of coronally advanced flap with or without connective tissue graft for the treatment of multiple adjacent gingival recessions in the aesthetic area: a randomized controlled clinical trial. *J Clin Periodontol* 43**,** 849-856.

Cairo, F., M. Nieri & U. Pagliaro (2014) Efficacy of periodontal plastic surgery procedures in the treatment of localized facial gingival recessions. A systematic review. *J Clin Periodontol* 41 Suppl 15**,** S44-62.

Chambrone, L., R.C.N. de Castro Pinto & L.A. Chambrone (2019a) The concepts of evidence-based periodontal plastic surgery: Application of the principles of evidence-based dentistry for the treatment of recession-type defects. *Periodontol 2000* 79**,** 81-106.

Chambrone, L., M.A.S. Ortega, F. Sukekava, R. Rotundo, Z. Kalemaj, J. Buti & G.P.P. Prato (2019b) Root coverage procedures for treating single and multiple recession-type defects: An updated Cochrane systematic review. *J Periodontol* 90**,** 1399-1422.

Chambrone, L., F. Sukekava, M.G. Araújo, F.E. Pustiglioni, L.A. Chambrone & L.A. Lima (2010) Root-coverage procedures for the treatment of localized recession-type defects: a Cochrane systematic review. *J Periodontol* 81**,** 452-478.

Chambrone, L. & D.N. Tatakis (2015) Periodontal soft tissue root coverage procedures: a systematic review from the AAP Regeneration Workshop. *J Periodontol* 86**,** S8-51.

Cortellini, P. & N.F. Bissada (2018) Mucogingival conditions in the natural dentition: Narrative review, case definitions, and diagnostic considerations. *J Clin Periodontol* 45 Suppl 20**,** S190-S198.

Dai, A., J.P. Huang, P.H. Ding & L.L. Chen (2019) Long-term stability of root coverage procedures for single gingival recessions: A systematic review and meta-analysis. *J Clin Periodontol* 46**,** 572-585.

Dodge, A., J. Garcia, P. Luepke, Y.L. Lai, M. Kassab & G.H. Lin (2018) The effect of partially exposed connective tissue graft on root-coverage outcomes: a systematic review and meta-analysis. *Eur J Oral Sci* 126**,** 84-92.

Goldstein, M., E. Nasatzky, J. Goultschin, B.D. Boyan & Z. Schwartz (2002) Coverage of previously carious roots is as predictable a procedure as coverage of intact roots. *J Periodontol* 73**,** 1419-1426.

Huang, L.H., R.E. Neiva & H.L. Wang (2005) Factors affecting the outcomes of coronally advanced flap root coverage procedure. *J Periodontol* 76**,** 1729-1734.

Hwang, D. & H.L. Wang (2006) Flap thickness as a predictor of root coverage: a systematic review. *J Periodontol* 77**,** 1625-1634.

Karam, P.S., A.C. Sant’Ana, M.L. de Rezende, S.L. Greghi, C.A. Damante & M.S. Zangrando (2016) Root surface modifiers and subepithelial connective tissue graft for treatment of gingival recessions: a systematic review. *J Periodontal Res* 51**,** 175-185.

Leknes, K.N., E.S. Amarante, D.E. Price, O.E. Bøe, R.J. Skavland & T. Lie (2005) Coronally positioned flap procedures with or without a biodegradable membrane in the treatment of human gingival recession. A 6-year follow-up study. *J Clin Periodontol* 32**,** 518-529.

McGuire, M.K., E.T. Scheyer & M.B. Snyder (2014) Evaluation of recession defects treated with coronally advanced flaps and either recombinant human platelet-derived growth factor-BB plus β-tricalcium phosphate or connective tissue: comparison of clinical parameters at 5 years. *J Periodontol* 85**,** 1361-1370.

Moisa, D.H., J.A. Connolly, B. Cheng & E. Lalla (2019) Impact of connective tissue graft thickness on surgical outcomes: A pilot randomized clinical trial. *J Periodontol* 90**,** 966-972.

Moslemi, N., M. Mousavi Jazi, F. Haghighati, S.P. Morovati & R. Jamali (2011) Acellular dermal matrix allograft versus subepithelial connective tissue graft in treatment of gingival recessions: a 5-year randomized clinical study. *J Clin Periodontol* 38**,** 1122-1129.

Pini Prato, G., R. Rotundo, D. Franceschi, F. Cairo, P. Cortellini & M. Nieri (2011) Fourteen-year outcomes of coronally advanced flap for root coverage: follow-up from a randomized trial. *J Clin Periodontol* 38**,** 715-720.

Pini Prato, G.P., C. Baldi, M. Nieri, D. Franseschi, P. Cortellini, C. Clauser, R. Rotundo & L. Muzzi (2005) Coronally advanced flap: the post-surgical position of the gingival margin is an important factor for achieving complete root coverage. *J Periodontol* 76**,** 713-722.

Pini Prato, G.P., D. Franceschi, P. Cortellini & L. Chambrone (2018a) Long-term evaluation (20 years) of the outcomes of subepithelial connective tissue graft plus coronally advanced flap in the treatment of maxillary single recession-type defects. *J Periodontol* 89**,** 1290-1299.

Pini Prato, G.P., C. Magnani & L. Chambrone (2018b) Long-term evaluation (20 years) of the outcomes of coronally advanced flap in the treatment of single recession-type defects. *J Periodontol* 89**,** 265-274.

Pini-Prato, G., C. Baldi, U. Pagliaro, M. Nieri, D. Saletta, R. Rotundo & P. Cortellini (1999) Coronally advanced flap procedure for root coverage. Treatment of root surface: root planning versus polishing. *J Periodontol* 70**,** 1064-1076.

Pini-Prato, G., D. Franceschi, R. Rotundo, F. Cairo, P. Cortellini & M. Nieri (2012) Long-term 8-year outcomes of coronally advanced flap for root coverage. *J Periodontol* 83**,** 590-594.

Rasperini, G., R. Acunzo, G. Pellegrini, G. Pagni, M. Tonetti, G.P. Pini Prato & P. Cortellini (2018) Predictor factors for long-term outcomes stability of coronally advanced flap with or without connective tissue graft in the treatment of single maxillary gingival recessions: 9 years results of a randomized controlled clinical trial. *J Clin Periodontol* 45**,** 1107-1117.

Rasperini, G., M. Codari, E. Limiroli, R. Acunzo, L. Tavelli & A.Z. Levickiene (2019) Graftless Tunnel Technique for the Treatment of Multiple Gingival Recessions in Sites with Thick or Very Thick Biotype: A Prospective Case Series. *Int J Periodontics Restorative Dent* 39**,** e203-e210.

Rasperini, G., M. Codari, L. Paroni, S. Aslan, E. Limiroli, C. Solís-Moreno, K. Suckiel-Papiór, L. Tavelli & R. Acunzo (2020) The Influence of Gingival Phenotype on the Outcomes of Coronally Advanced Flap: A Prospective Multicenter Study. *Int J Periodontics Restorative Dent* 40**,** e27-e34.

Santamaria, M.P., C.A. Silveira, I.F. Mathias, F.L.D.S. Neves, L.M. Dos Santos, M.A.N. Jardini, D.N. Tatakis, E.A. Sallum & E. Bresciani (2018) Treatment of single maxillary gingival recession associated with non-carious cervical lesion: Randomized clinical trial comparing connective tissue graft alone to graft plus partial restoration. *J Clin Periodontol* 45**,** 968-976.

Skurska, A., E. Dolińska, M. Sulewska, R. Milewski, J. Pietruski, S. Sobaniec & M. Pietruska (2015) The assessment of the influence of vertical incisions on the aesthetic outcome of the Miller class I and II recession treatment: a split-mouth study. *J Clin Periodontol* 42**,** 756-763.

Tatakis, D.N. & L. Chambrone (2016) The Effect of Suturing Protocols on Coronally Advanced Flap Root-Coverage Outcomes: A Meta-Analysis. *J Periodontol* 87**,** 148-155.

Tatakis, D.N., L. Chambrone, E.P. Allen, B. Langer, M.K. McGuire, C.R. Richardson, I. Zabalegui & H.H. Zadeh (2015) Periodontal soft tissue root coverage procedures: a consensus report from the AAP Regeneration Workshop. *J Periodontol* 86**,** S52-5.

Tavelli, L., S. Barootchi, F. Cairo, G. Rasperini, K. Shedden & H.L. Wang (2019a) The Effect of Time on Root Coverage Outcomes: A Network Meta-analysis. *J Dent Res* 98**,** 1195-1203.

Tavelli, L., S. Barootchi, R. Di Gianfilippo, M. Modarressi, F. Cairo, G. Rasperini & H.L. Wang (2019b) Acellular dermal matrix and coronally advanced flap or tunnel technique in the treatment of multiple adjacent gingival recessions. A 12-year follow-up from a randomized clinical trial. *J Clin Periodontol*

Tavelli, L., S. Barootchi, H. Greenwell & H.L. Wang (2019c) Is a soft tissue graft harvested from the maxillary tuberosity the approach of choice in an isolated site. *J Periodontol* 90**,** 821-825.

Tavelli, L., A. Ravidà, G.H. Lin, F.S. Del Amo, M. Tattan & H.L. Wang (2019d) Comparison between Subepithelial Connective Tissue Graft and De-epithelialized Gingival Graft: A systematic review and a meta-analysis. *J Int Acad Periodontol* 21**,** 82-96.

Zucchelli, G. & M. De Sanctis (2005) Long-term outcome following treatment of multiple Miller class I and II recession defects in esthetic areas of the mouth. *J Periodontol* 76**,** 2286-2292.

Zucchelli, G., I. Mounssif, C. Mazzotti, M. Stefanini, M. Marzadori, E. Petracci & L. Montebugnoli (2014) Coronally advanced flap with and without connective tissue graft for the treatment of multiple gingival recessions: a comparative short- and long-term controlled randomized clinical trial. *J Clin Periodontol* 41**,** 396-403.

Zucchelli, G., L. Tavelli, S. Barootchi, M. Stefanini, G. Rasperini, C. Valles, J. Nart & H.L. Wang (2019) The influence of tooth location on the outcomes of multiple adjacent gingival recessions treated with coronally advanced flap: A multicenter re-analysis study. *J Periodontol* 90**,** 1244-1251.

Zucchelli, G., L. Tavelli, A. Ravidà, M. Stefanini, F. Suárez-López Del Amo & H.L. Wang (2018) Influence of tooth location on coronally advanced flap procedures for root coverage. *J Periodontol* 89**,** 1428-1441.
