# Supplementary material for: Root coverage stability: A systematic overview of controlled clinical trials with at least 5 years of follow‐up
Source: Clin Exp Dent Res. 2021 Feb 9;7(5):692–710. doi: 10.1002/cre2.395 (PMC8543486; doi:10.1002/cre2.395)
Supplement: Supplementary file 9 — Appendix 9. Bubble plots that illustrate the relationship between the observational period in months (x‐axis) and the relative treatment effect observed in each trial (y‐axis) for each secondary outcome parameter. Each point refers to a trial. The size of each point has been weighted by the inverse of the variance of the corresponding trial: the smaller the trial, the smaller the size of the point and vice‐versa. Different colours refer to the comparisons investigated in each trial. A positive mean difference indicates that the second intervention in the comparison is more favorable. A bubble plot for CRC stability is not included due to lack of variance data in the original publications. [file CRE2-7-692-s010.pdf]

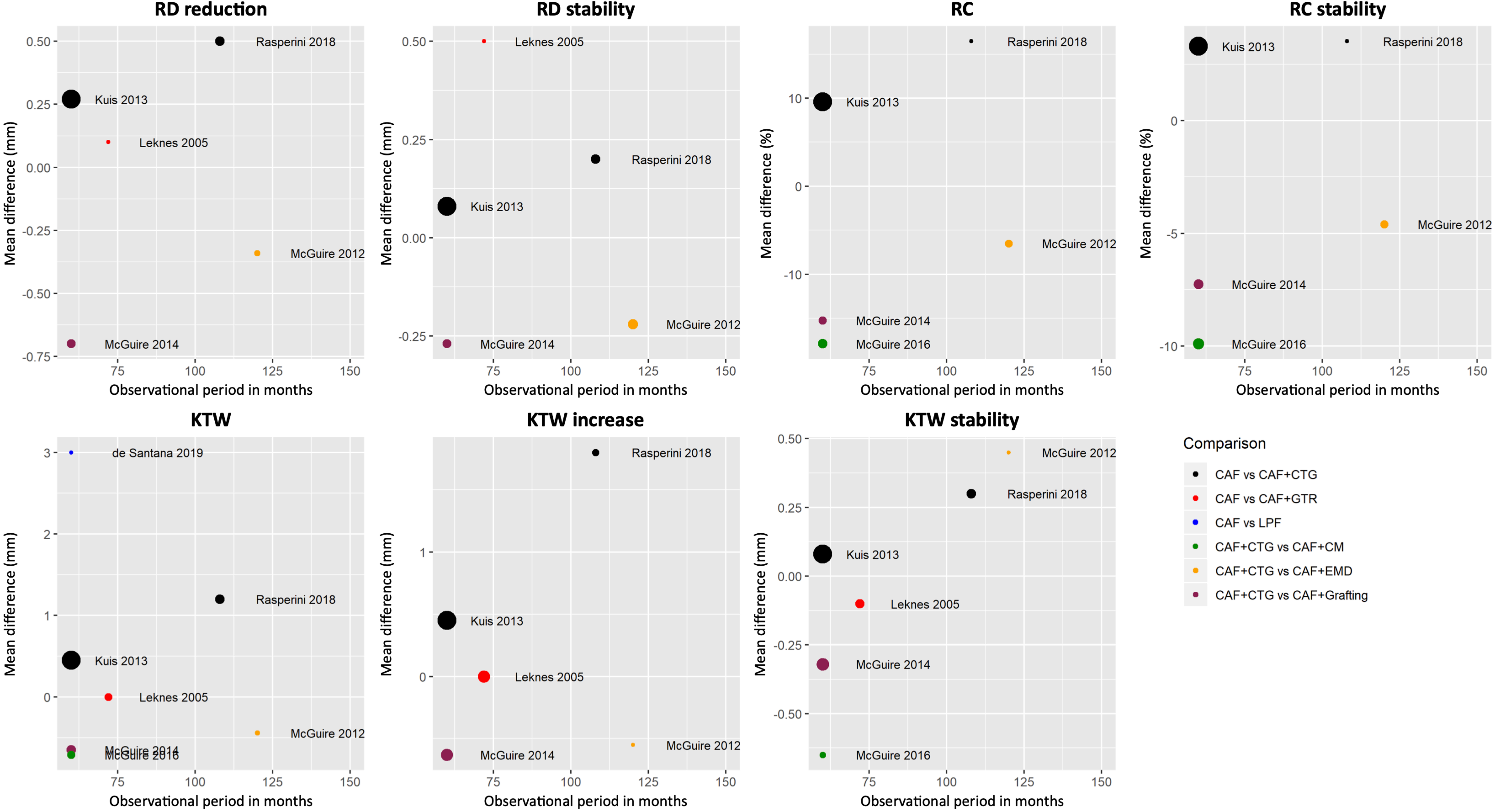
RD reduction
RD stability
RC
RC stability
KTW
KTW increase
KTW stability
Mean difference (mm)
Mean difference (%)
Observational period in months
Rasperini 2018
Kuis 2013
Leknes 2005
McGuire 2012
McGuire 2014
McGuire 2016
de Santana 2019
Comparison
CAF vs CAF+CTG
CAF vs CAF+GTR
CAF vs LPF
CAF+CTG vs CAF+CM
CAF+CTG vs CAF+EMD
CAF+CTG vs CAF+Grafting
