# Supplementary material for: Root coverage stability: A systematic overview of controlled clinical trials with at least 5 years of follow‐up
Source: Clin Exp Dent Res. 2021 Feb 9;7(5):692–710. doi: 10.1002/cre2.395 (PMC8543486; doi:10.1002/cre2.395)

**Appendix 10.** RoB assessment of the studies eligible for network meta-analysis applying the Cochrane Collaboration‘s Tool for assessing RoB Version 2; green represents a low risk, yellow some concerns, and red a high risk.

**a) Individual risk of studies eligible for network meta-analysis.**


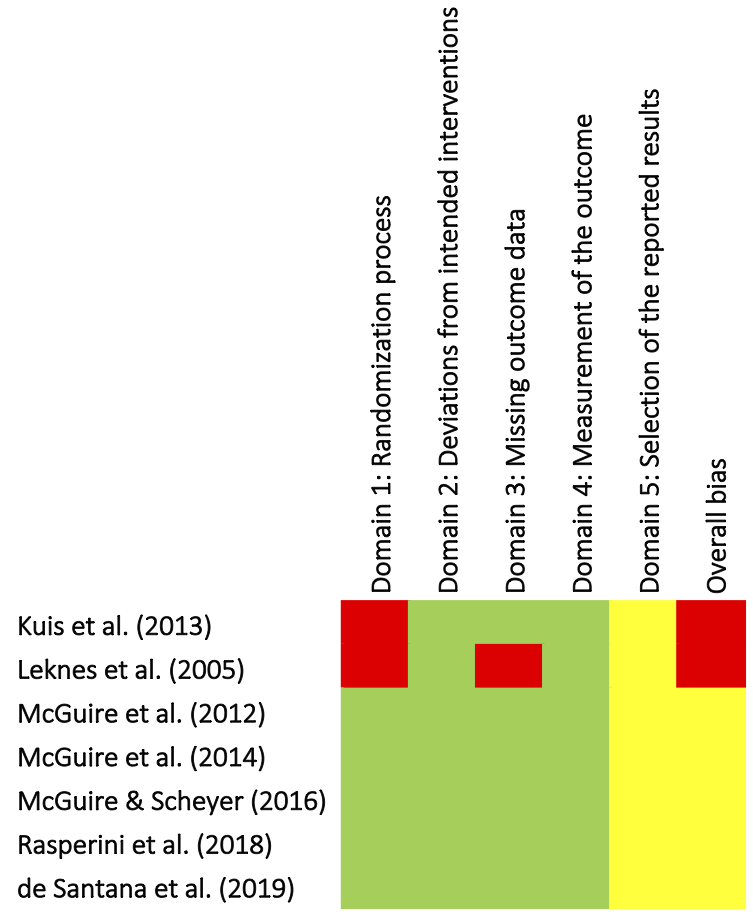


**b) Overall risk of studies eligible for network meta-analysis.**


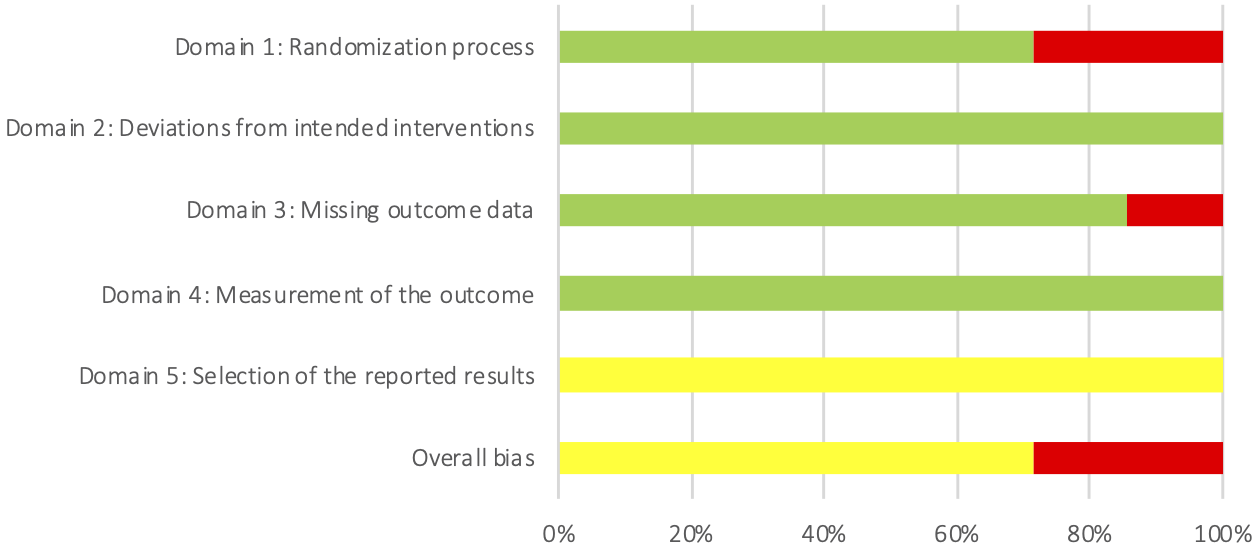

Supplement: Supplementary file 10 — Appendix 10. RoB assessment of the studies eligible for network meta‐analysis applying the Cochrane Collaboration's Tool for assessing RoB Version 2; green represents a low risk, yellow some concerns, and red a high risk. [file CRE2-7-692-s003.docx]
